# Neural Correlates of Balance Skill Learning in Young and Older Individuals: A Systematic Review and Meta-analysis

**DOI:** 10.1186/s40798-023-00668-3

**Published:** 2024-01-07

**Authors:** Lisanne B. M. Bakker, Claudine J. C. Lamoth, Tomas Vetrovsky, Markus Gruber, Simone R. Caljouw, Ward Nieboer, Wolfgang Taube, Jaap H. van Dieën, Urs Granacher, Tibor Hortobágyi

**Affiliations:** 1 Department of Human Movement Sciences, Center for Human Movement Sciences, University Medical Center Groningen, University of Groningen, A. Deusinglaan 1, 9700 AD Groningen, The Netherlands; 2 Faculty of Physical Education and Sport, Charles University, Prague, Czech Republic; 3 Department of Sport Science, Human Performance Research Centre, University of Konstanz, Constance, Germany; 4 Department of Neurosciences and Movement Sciences, University of Fribourg, Fribourg, Switzerland; 5 Department of Human Movement Sciences, Amsterdam Movement Sciences, Vrije Universiteit Amsterdam, Amsterdam, The Netherlands; 6 Department of Sport and Sport Science, Exercise and Human Movement Science, University of Freiburg, Freiburg, Germany; 7 Department of Kinesiology, Hungarian University of Sports Science, Budapest, Hungary; 8 Institute of Sport Sciences and Physical Education, University of Pécs, Pecs, Hungary; 9 Somogy County Kaposi Mór Teaching Hospital, Kaposvár, Hungary

**Keywords:** Healthy aging, Postural balance, Balance training, Neural adaptation

## Abstract

**Background:**

Despite the increasing number of research studies examining the effects of age on the control of posture, the number of annual fall-related injuries and deaths continues to increase. A better understanding of how old age affects the neural mechanisms of postural control and how countermeasures such as balance training could improve the neural control of posture to reduce falls in older individuals is therefore necessary. The aim of this review is to determine the effects of age on the neural correlates of balance skill learning measured during static (standing) and dynamic (walking) balance tasks in healthy individuals.

**Methods:**

We determined the effects of acute (1–3 sessions) and chronic (> 3 sessions) balance skill training on balance in the trained and in untrained, transfer balance tasks through a systematic review and quantified these effects by robust variance estimation meta-analysis in combination with meta-regression. We systematically searched PubMed, Web of Science, and Cochrane databases. Balance performance and neural plasticity outcomes were extracted and included in the systematic synthesis and meta-analysis.

**Results:**

Forty-two studies (*n* = 622 young, *n* = 699 older individuals) were included in the systematic synthesis. Seventeen studies with 508 in-analysis participants were eligible for a meta-analysis. The overall analysis revealed that acute and chronic balance training had a large effect on the neural correlates of balance skill learning in the two age groups combined (*g* = 0.79, *p* < 0.01). Both age groups similarly improved balance skill performance in 1–3 training sessions and showed little further improvements with additional sessions. Improvements in balance performance mainly occurred in the trained and less so in the non-trained (i.e., transfer) balance tasks. The systematic synthesis and meta-analysis suggested little correspondence between improved balance skills and changes in spinal, cortical, and corticospinal excitability measures in the two age groups and between the time courses of changes in balance skills and neural correlates.

**Conclusions:**

Balance skill learning and the accompanying neural adaptations occur rapidly and independently of age with little to no training dose-dependence or correspondence between behavioral and neural adaptations. Of the five types of neural correlates examined, changes in only spinal excitability seemed to differ between age groups. However, age or training dose in terms of duration did not moderate the effects of balance training on the changes in any of the neural correlates. The behavioral and neural mechanisms of strong task-specificity and the time course of skill retention remain unclear and require further studies in young and older individuals.

*Registration*: PROSPERO registration number: CRD42022349573.

**Supplementary Information:**

The online version contains supplementary material available at 10.1186/s40798-023-00668-3.

## Key Points

- We examined the effects of balance training on balance performance, and its neural correlates in healthy younger (*n* = 21 studies) and older individuals (*n* = 18 studies)
- Balance training improves the trained balance skill rapidly, i.e., 30–40 min
- Balance training improves the non-trained balance skills (i.e., transfer tasks) much less or not at all
- Balance training improves neural measures independently of age and training dose
- Age and training duration do not moderate the training-induced changes in the neural correlates of balance learning
- There is little-to-no temporal correspondence between improvements in balance skills and the neural correlates examined

**Supplementary Information:**

The online version contains supplementary material available at 10.1186/s40798-023-00668-3.

## Introduction

Postural control is the ability to align the body’s position in space for the purpose of balance and orientation and is necessary to perform activities of daily living [1]. The ability to control posture determines static (standing) and dynamic (walking) balance performance [2–4]. A historical understanding of voluntary movement already assigned a putative role to the neural control of posture [5]. Therefore, it is no surprise that age-related impairments in the neural control of posture increase the risk of losing balance and falls [6–8]. Nearly 40% of older individuals aged over 65, even if apparently healthy, experience at least one fall annually [8, 9]. Curiously while the last three decades saw an increasing number of research studies examining the effects of age on the control of posture (up to ~ 220 studies per year), the number of annual fall-related injuries and deaths continued to increase [10]. Such data highlight the need to improve our understanding of how old age affects the muscular [11, 12] and the neural mechanisms of postural control. Moreover, how countermeasures such as balance training could improve the neural control of posture to reduce falls in older individuals [2, 3, 8].

The ability to control posture is a complex skill involving multiple degrees of freedom and is based on the interaction among dynamic sensorimotor processes [13]. Akin to manual motor skills [14], skills involved in postural control can improve rapidly in healthy adults, by ~ 50% after practicing the skill just for ~ 20 min [15], i.e., acute (1–3 sessions) balance training. After further practice (i.e., chronic: > 3 balance training sessions), the balance skill is consolidated into memory and retained for weeks [16] or even months [17]. In line with the learning of other motor skills, the learning of balance skills is also highly task-specific [18] and can be subject to motor interference [19].

However, there are gaps in our understanding of the behavioral and neural mechanisms underlying balance skill learning and how old age affects these processes. First, manual motor skill learning evidence suggests that while age may affect motor performance, age does not necessarily affect the magnitude and especially the rate of skill improvement following motor practice [20, 21]. However, simple experimentally controlled manual tasks are very different from whole-body balance tasks. A balance skill involves other systems in addition to the ones operative in manual skill acquisition (vestibular system, for example). In addition, a balance skill involves many more degrees of freedom than a single joint manual skill, making it likely that the time course of balance skill learning is different. Whether this is the case is currently unclear. Second, the practice of a manual skill seems to plateau after a few sessions, even in older individuals [14]. An exploratory perusal of balance skill learning studies suggests little [16]-to--no-dose effects of practice frequency (i.e., session number) on balance learning [22], implying a possible plateauing of balance skill performance in line with manual skill learning. Whether the number of exercise sessions is related to the retention of the trained balance skill is unresolved. Knowledge of the retention of the balance skill after skill acquisition is essential to determine whether the skill is consolidated into motor memory [23], our exploratory search indicated a paucity of retention data.

Furthermore, it is unclear whether the plateauing of balance skill performance after acute balance training is accompanied by a parallel leveling off of adaptations in neural correlates and whether such effects would occur independently of age. A parallel leveling off of neural adaptations would be an important issue because it has relevance for the prescription and personalization of balance training by individually adjusting the difficulty and complexity of balance exercise, training frequency, and volume to move participants off the adaptation plateau. Finally, a hallmark of motor skill learning is specificity: there is little to no transfer of the just-acquired skill to skills that mimic the trained task but were not practiced [18]. When the spatiotemporal characteristics of these transfer tasks differ from those in the trained task, the specificity of testing is affected, and balance skill learning might be under or overestimated, which could have implications for fall prevention [8].

It is well established that neuroplasticity, i.e., the brain’s ability to create new and reorganize existing synaptic connections [24–26], underlies manual motor skill acquisition, retention, and transfer [14]. Several studies reported adaptations in spinal [27–30], corticospinal [29–32], motor cortical [33–36], and brain network [17, 35, 37] mechanisms following balance skill learning. Spinal correlates of balance learning involve the H-reflex measured in different postures, at rest, and during activation of the target muscle to a certain level [27–30]. Corticospinal correlates of balance skill learning comprise responses to magnetic brain stimulation alone or in combination with peripheral nerves stimulation [29–32]. Motor cortical correlates of balance skill learning are indexed by magnetic brain stimulation involving double pulse paradigms and imaging [38–40]. Imaging is also used to determine the effects of balance skill training on functional, structural, and network markers of neuroplasticity [17, 35, 37]. However, if and how age affect these neural correlates of balance skill learning have not been systematically and meta-analytically examined. Therefore, the overall aim of this systematic review and meta-analysis was to determine the effects of age on the neural correlates of balance skill learning measured during static (standing) and dynamic (walking) balance tasks. Specifically, we focused on the effects of acute (1–3 balance training sessions) and chronic balance training (> 3 balance training sessions) on the neural correlates of balance skill acquisition, transfer (specificity), and retention. Based on the data in the manual motor skill learning literature [14, 41], our working hypotheses were that both young and older individuals would: (1) acquire balance skills rapidly during acute balance training with little further improvements with ongoing chronic balance training; (2) improve balance performance mainly in the trained and less so in the non-trained balance tasks (i.e., transfer specificity), and that (3) age would have minor effects on the neural correlates of balance skill learning. Due to a lack of data on balance skill retention in either age group, currently, we are unable to propose a hypothesis concerning the effects of age on balance skill retention.

## Methods

### Review Design

This is a systematic review and meta-analysis conducted in accordance with the Preferred Reporting Items for Systematic Reviews and Meta-Analysis (PRISMA) statements [42] and registered at PROSPERO (CRD42022349573).

### Search Strategy

After consultation with a review expert librarian, three electronic databases were searched (Cochrane Library, PubMed, Web of Science) up to July 1, 2023, using the following database-specific main terms: a) Postural balance or gait, b) neural correlates, and c) balance training. With the understanding that balance training is the most often used yet inconsistent term, we defined balance training as an exercise regime that incorporates exercises that perturb or displace the center of mass or base of support intending to improve postural stability [43]. Therefore, in line with previous balance training reviews [43, 44], we identified balance training programs with the following search terms: “balance training” OR “neuromuscular training” OR “proprioceptive training” OR “sensorimotor training” OR “instability training” OR “perturbation training”. During a preliminary search using these terms, we encountered studies that implemented Tai Chi, dance training, and Yoga as balance interventions. Therefore, we added these interventions to our search term. In this review, we will use the term balance training to describe any training program primarily aimed at improving postural control and balance performance, regardless of the terms used in the studies. Additional file 1: Fig. S1 provides the full electronic search strategy used in PubMed.

### Study Selection

We included all studies published in peer-reviewed journals and reported in English if the study: (a) examined a group of healthy individuals aged ≥ 18 years; (b) included a balance training protocol aimed at improving either static or dynamic balance; and (c) tested at least one measure of neural adaptation (e.g., cortical, corticospinal, spinal excitability, blood markers, functional and structural brain adaptations). Studies with the following characteristics were excluded: (a) no balance training, (b) no measure of neural adaptation, (c) only patient groups or populations with adverse health events, (d) review paper, (e) study protocol, or (f) case study. Rayyan was used to identify and remove duplicates from the resulting studies. Based on the defined inclusion and exclusion criteria, two authors independently screened titles and abstracts of potentially relevant studies (LB, WN). If still potentially relevant after initial screening, two authors independently screened full texts (LB, WN) to examine eligibility. Any discrepancies in the selection process were resolved through consultation with the other authors. Studies explicitly designed to down-train the H-reflex and not to improve balance were not included in this review. The resulting studies were included in a systematic synthesis. In addition, all randomized controlled trials in which the control intervention did not aim to improve postural control were included in a meta-analysis.

### Data Extraction and Coding

Two authors extracted and synthesized data into tables (LB, WN). Studies were coded for: first author, year of publication, participant characteristics (age, sex, number of participants), balance intervention type, duration, and frequency, the total number of training sessions, and balance, gait, and neural adaptation outcomes. In the case of data reported only in charts and plots, semi-automated software was used to extract data from *x*–*y* plots (http://getdata-graph-digitizer.com/). When data were not available in the article, corresponding authors were contacted to request the data.

While there are other definitions and means to categorize balance tasks [45, 46], this review, in line with previous studies [47, 48], structured static and dynamic test task conditions based on the goal of the task: static balance consisting of perturbed or unperturbed upright standing and dynamic balance consisting of perturbed or unperturbed walking. The population was categorized as healthy young individuals if the mean group age was between 18 and 30 years and as healthy older individuals if the mean group age was over 65 years old. Studies with a mean group age between 30 and 65 were not included in the meta-analysis. The duration of the balance intervention was categorized as acute when the intervention consisted of 1–3 sessions over a period of days and chronic when the intervention consisted of > 3 sessions over a period of weeks or months. Neural adaptation outcomes were categorized as spinal excitability, corticospinal excitability, brain structure, and neurochemical outcomes.

### Assessment of Methodological Quality

Two authors (LB and TH) independently assessed the methodological quality of included studies using the Physiotherapy Evidence Database (PEDro) Scale. Each of the ten criteria was independently graded ‘1’ for ‘yes’ and ‘0’ for ‘no’. Disagreements were resolved with discussions between the authors. PEDro scores of 0–3, 4–5, 6–8, and 9–10 were, respectively, considered ‘poor’, ‘fair’, ‘good’, and ‘excellent’.

### Statistical Analyses

Statistical analyses were performed in R (version 4.2.1.) using robumeta (version 2.0). metafor (version 3.8–1), and clubSandwich (version 0.5.8) packages. A random-effect meta-regression method called robust variance estimation for multilevel data structures was used to pool the data, allowing for the inclusion of multiple dependent outcome measures from the same study. Robust variance estimation assesses the variance of meta-regression coefficient estimates using the observed residuals and does not require weight or distributional assumptions [49, 50]. To account for the correlated effects within the studies, ‘study’ was used as the clustering variable. The observations were weighted with the inversion of the sampling variance. First, we computed the overall summary effects of all included studies for balance performance and neural adaptations and visualized them using forest plots. Because the current review was focused on papers including a measure of neural adaptations, thus ignoring potentially hundreds of balance training studies, we performed the meta-analysis on balance performance outcomes only to characterize the sample of our studies, strictly avoiding any generalization. We performed sensitivity analyses for the effects of balance training on neural adaptations by assessing how influential cases affected the results. The influential cases were diagnosed using a combination of methods (externally standardized residuals, difference in fits values, Cook's distances, covariance ratios, leave-one-out estimates of the amount of heterogeneity, leave-one-out values of the test statistics for heterogeneity, hat values, weights) as implemented in the 'influence' function within the metafor package [51]. We also examined potential publication bias using funnel plots supplemented with Egger's regression test [52]. For all analyses, we computed Hedges’ g effect size (*g* = 0.15, 0.40, 0.75, respectively, small, medium, and large effects) [53], standard error (SE) of the effect size, 95% confidence intervals, the statistical significance of the effect (set at *p* < 0.05), percentage of heterogeneity (*I*^2^), and the absolute value of true heterogeneity (Tau^2^). The values of *I*^2^ > 25%, > 50%, and > 75% indicate, respectively, low, moderate, and high heterogeneity [54, 55]. The direction of effect sizes was standardized so that positive effect sizes would reflect improvements in a given outcome. For example, an increase in the numerical value of SICI after balance training was coded as a positive change because an improvement in balance performance is thought to be accompanied by an increase in cortical inhibition. In contrast, an increase in the H-reflex was coded as negative because an improvement in balance performance is thought to be accompanied by a reduction in the H-reflex. Second, to explore potential effect moderators, we assessed age group, training duration, and neural adaptation outcomes as covariates. The statistical significance of categorical moderators with three or more levels was tested using the Hotelling–Zhang test. Thirdly, we performed subgroup meta-analyses for different age groups, training durations, and neural adaptation outcome measures. Finally, to examine whether balance training induced changes in neural adaptation were associated with changes in balance performance (i.e., center of pressure velocity, time in balance, gait speed), we performed a random-effects meta-regression, treating the neural adaptation effect sizes as the outcome and the balance performance outcome effect sizes as the continuous predictors.

## Results

### Study Selection

**Fig. 1:**
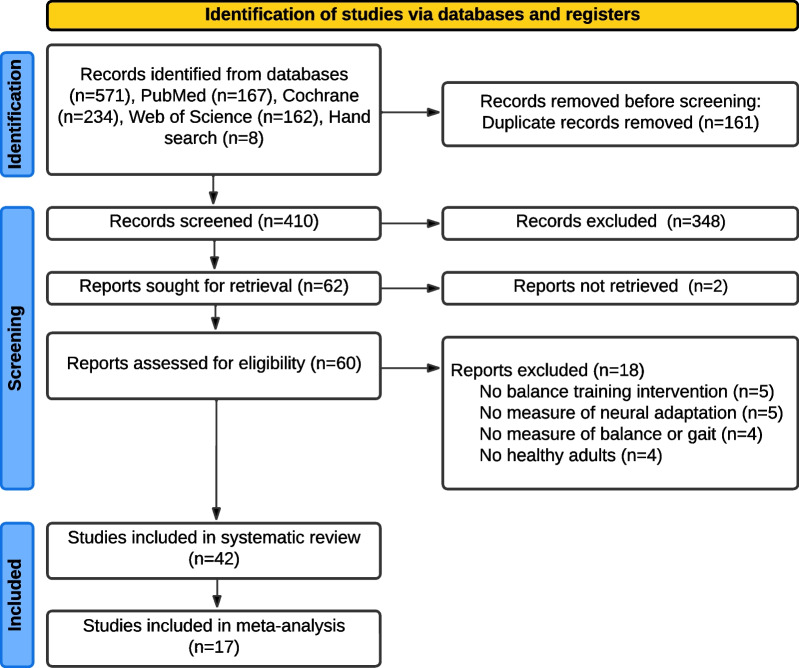
PRISMA flowchart of search and study selection

Figure 1 shows the PRISMA flowchart of the search and study selection. After duplicates were removed, the search resulted in 410 articles published upto July 1, 2023. Title and abstract screening resulted in 62 articles of interest, of which 42 were identified for inclusion.

### Description of the Included Studies

#### Study Characteristics

Table 1 summarizes the 42 included studies, of which 22 examined healthy young individuals (*n* = 622, 311 males, age: 26 ± 4) and 20 healthy older individuals (*n* = 699, 331 males, age: 68 ± 5). Sample sizes ranged from 8 to 54 participants in the control groups and 8 to 47 participants in the balance training groups. Of the 42 included studies, 17 randomized controlled trials were included in the meta-analysis.

**Table 1:** Characteristics of included studies, participants, interventions, and outcome measures

Study	Participant characteristics			Intervention		Outcome measures	
	Group	N (male)	Age	Type	Duration	Balance and gait performance	Neural adaptation
*Studies examining the effects of balance training on spinal excitability in healthy young individuals*							
Giboin [63]	HY-BT	18(8)	27 ± 8	BT: unipedal stance on floor and tilt board	1 day	Time in balance during unipedal stance on tiltboard, hands akimbo	SOL H-reflex amplitude normalized to Mmax at rest, bipedal stance on floor/board
Freyler [27]	HY-BT 1 HY-BT 2	16(8) 16(7)	24 ± 2 24 ± 2	BT partially unloaded balance exercises BT 2: mix of balance exercises	35 min, 2/wk, 4wks	CoP displacement in AP and ML direction during uni/bipedal stance on stable and unstable surfaces, hands akimbo	SOL H-reflex amplitude during stable surface uni/bipedal and unipedal unstable surface stance
Keller [65]	HY-BT HY-CON	12(6) 12(6)	24 ± 1	BT: Slackline standing and walking, increasing difficulty CON: no intervention	90 min, 2–3/wk, 4wks	Sway path during bipedal EO stabilization of Posturomed following perturbation and slackline standing	SOL H/M-ratios during bipedal quiet stance and unipedal stance on cushion, Posturomed and slackline
Giboin [68]	HY-BT Y-CON	22(12) 2(8)	25 ± 4 22 ± 2	BT: Slackline standing and walking CON: no intervention	45 min, 2/wk, 6wks	Number of steps on slackline and time in balance during unipedal stance on tiltboard, hands akimbo	SOL H-reflex amplitude normalized to Mmax assessed stepping onto slackline/ tiltboard
Schubert [31]	HY- BT HY-ST HY-CON	14(8) 12(7) 11(7)	26 ± 3 27 ± 6 28 ± 5	BT: Unipedal stance on different devices ST: Plantar extensions and dorsal flexions ankle joint CON: no intervention	45–55 min, 4/wk, 4wks	Ground reaction forces directed to compensate backwards perturbation	SOL Hmax/Mmax during dynamic plantar flexion/ perturbation and quiet standing
Gruber [28]	HY-BT HY-ST HY-CON	11(7) 9(4) 9(5)	25 ± 3 26 ± 5 26 ± 3	BT: unipedal EO stance on different devices ST: explosive ankle strength training CON: no intervention	60 min, 4/wk, 4wks	Sway path during unipedal stance on Posturomed, hands akimbo	SOL Hmax/Mmax during seated rest
Taube [29]	HY-BT HY-CON	13(8) 10(4)	25 ± 3 27 ± 5	BT: unipedal stance on different devices CON: no intervention	60 min, 4/wk, 4wks	Ground reaction forces directed to compensate perturbation, sway path on platform	SOL Hmax/Mmax during short and long latency response to backwards perturbation
Beck [30]	HY-BT HY-ST HY-CON	9(6) 8(4) 10(7)	28 ± 2 28 ± 2 26 ± 1	BT: unipedal stance on different devices ST: ballistic ankle strength training CON: no intervention	60 min, 4/wk, 4wks	Sway path during unipedal EO stance on Posturomed, hands akimbo	SOL and TA Hmax/Mmax during plantarflexion, backward perturbation, and rest
Behrens [64]	HY-BT HY-CON	13(7) 13(7)	25 ± 3 23 ± 2	BT: uni- and bipedal stance on different devices, increasing difficulty CON: no intervention	60 min, 2/wk, 8wks	CoP sway in AP and ML direction during bipedal stance EO on rigid and foam surface, hands akimbo	SOL Hmax/Mmax at rest and during MVC
*Studies examining the effects of balance training on spinal excitability in healthy older individuals*							
Alizadehsaravi [16]	HO-BT	22(11)	73 ± 4	BT: Mix of unipedal exercises	1 × 30-min and 10 × 45-min, 3wks	Balance robustness: mean time in balance on a balance platform, CoM velocity in ML direction	H-reflex and paired-pulse depression
Penzer [67]	HO-ST HO-BT HO-CON	10(2) 8(2) 8(2)	71 ± 6 71 ± 6 70 ± 6	ST: leg strength exercises BT: uni- and bipedal EO and EC stance on different devices CON: no intervention	60 min, 2/wk, 6wks	CoP velocity during bipedal EO/EC standing on unstable surface	SOL Hmax/Mmax and Hslope during rigid EO bipedal standing
Ruffieux [66]	HO-BT HO-CON	15(7) 13(8)	70 ± 4 71 ± 5	BT: unipedal stance on different devices CON: no intervention	3/wk, 5wks	CoP sway and # of errors in uni/bipedal stance on stable surface and unstable platform	SOL Hmax/Mmax during quiet stance
Lauber [69]	HO-BT HO-CON	13(7) 10(5)	67 ± 2 68 ± 4	SKI: alpine skiing CON: no intervention	28.5 days over 12wks	Postural sway while bipedal EO standing and while standing on a spinning top	SOL Hmax/Mmax during stable and unstable bipedal stance
Chen [70]	HO-BT HO-CON	20(11) 14(6)	73 ± 4 73 ± 7	BT: Tai Chi@CON: no intervention	60 min, 3/wk, 12wks	CoP displacement during bipedal EO and EC stance on a stable and unstable surface	SOL Hmax/Mmax during bipedal EO and EC stance on a stable and unstable surface
*Studies examining the effects of balance training on corticospinal excitability in healthy young individuals*							
Bakker [39]	HY-BT HY-CT HY-CON	12(6) 12(6) 12(6)	21 ± 1 21 ± 2 22 ± 3	BT: bipedal stance wobble board CT: stationary cycling CON: no intervention	1 × 20 min	Time in balance on wobble board, ST and DT beam walking on wide and narrow beam, and CoP velocity during bipedal EO/EC stance on rigid and foam surface in wide and narrow stance	TMS: MEP, SICI, LICI, ICF in TA during sitting and standing
Mouthon [73]	HY-BT HY-CON	13(9) 13(9)	24 ± 3	BT: Bipedal stance on movable platform CON: no intervention	45 min, 3/wk, 2wks	Time in balance on stable, semi-stable and unstable device, bipedal EO stance	TMS: MEP and SICI in TA and SOL during bipedal EO stance on stable, semi-stable and unstable device
Taube [71]	HY-ST HY-BT	24(14)	23 ± 2	ST: explosive strength training BT: unipedal stance on different devices, increasing difficulty	60 min, 3/wk, 4wks	Sway path during unipedal quiet stance and recovery following AP perturbations	TMS: SICI in SOL and TA during plantar/dorsiflexion, balance perturbations or at rest
Lauber [72]	HY-BT-ST HY-ST-BT	12(5) 12(5)	23 ± 2	ST: leg strength exercises BT: unipedal EO stance on different devices, increasing difficulty	3/wk, 4wks	Platform sway during unipedal stance on Posturomed when standing still and post perturbation	TMS: MEP and SICI in SOL during dynamic plantarflexions, balance perturbations and rest
Schubert [31]	HY- BT HY-ST HY-CON	14(8) 12(7) 11(7)	26 ± 3 27 ± 6 28 ± 5	BT: Unipedal stance on different devices ST: Plantar extensions and dorsal flexions ankle joint CON: no intervention	45–55 min, 4/wk, 4wks	Ground reaction forces directed to compensate backwards perturbation	Conditioned SOL H-reflex during dynamic plantar flexion/ backwards perturbation and quiet standing
Taube [29]	HY-BT HY-CON	13(8) 10(4)	25 ± 3 27 ± 5	BT: unipedal stance on different devices CON: no intervention	60 min, 4/wk, 4wks	ground reaction forces directed to compensate perturbation, sway path on platform	TMS: MEP and conditioned H-reflex in SOL during short and long latency response to backwards perturbation
Beck [30]	HY-BT HY-ST HY-CON	9(6) 8(4) 10(7)	28 ± 2 28 ± 2 26 ± 1	BT: unipedal stance on different devices ST: ballistic ankle strength training CON: no intervention	60 min, 4/wk, 4wks	Sway path during unipedal EO stance on Posturomed, hands akimbo	TMS: During plantarflexion, backward perturbation, and rest: MEP, SICI and ICF in SOL and TA
*Studies examining the effects of balance training on corticospinal excitability in healthy older individuals*							
Penzer [67]	HOA-ST HOA-BT HO-CON	10(2) 8(2) 8(2)	71 ± 6 71 ± 6 70 ± 6	ST: leg strength exercises BT: uni- and bipedal EO and EC stance on different devices CON: no intervention	60 min, 2/wk, 6wks	CoP velocity during bipedal EO/EC standing on unstable surface	TMS: MEP and MEP slope in SOL during rigid EO bipedal standing
Ruffieux [66]	HO-BT HO-CON	15(7) 13(8)	70 ± 4 71 ± 5	BT: unipedal stance on different devices CON: no intervention	3/wk, 5wks	CoP sway and # of errors in uni/bipedal stance on stable surface and unstable platform	TMS: SOL conditioned H-reflex during anterior posterior perturbation
Esculier [32]	HO-BT PD-BT	8(5) 8(5)	62 ± 12 64 ± 12	BT: Wii-fit with balance and squat exercises	40 min, 3/wk, 6wks	Unipedal EO stance time, CoP RMS velocity and dynamic balance tasks	TMS: MEP in QF and SOL during AO, imagery, and imitation
*Studies examining the effects of balance training on cortical activity in healthy young individuals*							
Peterson [75]	HY VR1 HY-VR2 HY-BT	10(5) 10(5) 10(5)	23 ± 5	Heel to toe beam walking, visual conditions: (1) VR + pert., (2) VR no pert., (3) unaltered view	1 × 30 min	# of beam walking errors	EEG: whole brain theta, alpha, beta and gamma power during beam walking
Zandvoort [74]	HY-BT	15(11)	24.7 ± 3.1	Unipedal stance on unstable boards, increasing difficulty	1 × 30 min	Time to recovery following a platform perturbation, allowed to use arms	EEG: Cortical source localization, cortico synergy connectivity following a perturbation
Ueta [56]	HY-BT-AT HY-AT-BT	14 14	22 ± 1	BT: slackline training AT: stationary cycling	1 × 30 min + after 4 weeks 1 × 30 min	Slackline standing and walking, unipedal EC standing on floor and balance disk, star excursion test	fMRI at rest: functional connectivity
Patel [76]	HY-BT	10	27 ± 4	BT: Treadmill-Slip perturbation training	3 days	# of compensatory steps and COM stability following treadmill perturbation	fMRI: activity during motor imagery of slipping and walking tasks
Taubert [33]	HY-BT HY-CON	28(14)	26 ± 3	BT: bipedal standing on unstable device	45 min, 1/wk, 6wks	Time in balance on unstable device, bipedal EO stance	fMRI at rest: functional connectivity
Giboin [68]	HY-BT HY-CON	22(12) 22(8)	25 ± 4 22 ± 2	BT: Slackline standing and walking	45 min, 2/wk, 6wks	Number of steps on slackline and time in balance during unipedal stance on tiltboard, hands akimbo	fMRI at rest: functional connectivity
*Studies examining the effects of balance training on cortical activity in healthy older individuals*							
Ruffieux [40]	HO-BT HO-CON	15(7) 12(7)	70 ± 4 72 ± 5	BT: One leg BT on unstable devices CON: no training	60 min, 3/wk, 5wks	# of errors in unipedal stance on stable and unstable platform	fMRI: activity during motor imagery and action observation + motor imagery
Magon [35]	HO-BT HO-CON	14(8) 14(8)	62 ± 5 62 ± 5	BT: slackline standing and walking, increasing difficulty CON: educational fall prevention sessions	30 min, 3/wk, 6wks	Slackline stance time in unipedal and tandem EO stance	fMRI at rest: functional connectivity
Adcock [59]	HO-BT	19(9)	71 ± 6	Exergame based on Tai Chi and dance	40 min, 3/wk, 7wks	Gait speed in single and dual-task walking, balance score extended SPPB	EEG: Cortical activity during resting state, ROI: parieto-occipital
Eggenberger [36]	HO-BT HO-DT	14(5) 19(7)	78 ± 7 73 ± 6	BT: uni/bipedal stance on different devices DT: interactive video game-based dancing	30 min, 3/wk, 8wks	Balance score extended SPPB	fNIRS: cortical activity during walking at preferred and fast speed, ROI: PFC
Kubica [34]	HO-BT1 HO-BT2 HO-CON	28(13) 15(9) 23(14)	66 ± 1 65 ± 1 64 ± 1	BT: mix of balance exercises BT: Wii Fit Balance board CON: no intervention	30/60 min, 3/wk, 12wks	POMA	fMRI: during motor imagery and/or action observation
Chen [77]	HO-BT HO-CON	12 15	68 ± 1 70 ± 1	BT: Tai Chi CON: health lectures	60 min, 4/w, 16wks	Gait speed (m/s) during negotiating obstacle task with and without an added cognitive task	fNIRS: cortical activity during negotiating obstacle task, ROI: PFC
*Studies examining the effects of balance training on structural brain adaptations in healthy young individuals*							
Ueta [56]	HY-BT-AT HY-AT-BT	14 14	22 ± 1	BT: slackline training AT: stationary cycling	1 × 30 min + after 4 weeks 1 × 30 min	Slackline standing and walking, unipedal EC standing on floor and balance disk, star excursion test	MRI: gray matter volume and WM microstructure
Taubert [22]	Exp. 1: HY-BT HY-CON1 Exp 2: HY-BT Exp 3: HY-CON2	21(10) 16(8) 11(4) 11(5)	26 ± 4 26 ± 4 26 ± 3 28 ± 3	BT: bipedal standing on unstable device CON1: seated rest CON2: motor action control task: repetitive high knees	Exp. 1: 1 × 45 min Exp 2: BT: 1 day, 3 × 20 min Exp 3: 1 × 45 min	Time in balance on unstable device, bipedal EO stance	MRI: cortical thickness and gray matter density
Taubert [37]	HYA-BT HY-CON	28(14)	26 ± 3	BT: bipedal standing on unstable device	45 min, 1/wk, 6wks	Time in balance on unstable device, bipedal EO stance	MRI: gray matter volume and structural connectivity: fractional anisotropy in white matter regions
Im [57]	HA-BT	17(11)	47 ± 13	BT: home based mix of bipedal and uni/bipedal stance on different devices	30 min, 3/wk, 4wks	BBS and Community Balance and Mobilty Scale	MRI: whole brain fractional anisotropy
Rogge [78]	HA-BT HA-CON	19 (7) 18 (7)	44 ± 15 46 ± 15	BT: uni- and bipedal EO and EC stance on different devices, increasing difficulty CON: relaxation training	50 min, 2/wk, 12wks	Time in balance during bipedal EO stance on unstable platform, hands akimbo	MRI: cortical thickness and gray matter volume, ROI: hippocampus and basal ganglia
*Studies examining the effects of balance training on structural brain adaptations in healthy older individuals*							
Sehm [17]	PD-BT HO-BT	20(11) 16(9)	65 ± 2 63 ± 2	BT: bipedal stance unstable platform	~ 45 min, 1/wk, 6wks	Time in balance on unstable platform	MRI: whole brain gray matter volume
Burciu [60]	CD-BT H-BT	19(13) 19(11)	56 ± 12 54 ± 13	BT: move COG to target during bipedal stance on stable and unstable platform, increasing difficulty	30/60 min, 7/wk, 2wks	Gait speed, postural sway, COG displacement velocity during bipedal EO and EC on a stable and unstable platform, BBS and TUG	MRI: whole brain gray matter volume
Magon [35]	HO-BT HO-CON	14(8) 14(8)	62 ± 5 62 ± 5	BT: slackline standing and walking, increasing difficulty CON: educational fall prevention sessions	30 min, 3/wk, 6wks	Slackline stance time in unipedal and tandem EO stance	MRI: brain volume
Adcock [58]	HO-BT HO-CON	15(5) 16(10)	77 ± 6 71 ± 5	BT: Exergame based on Tai Chi and dance CON: no intervention	30-40 min, 3/wk, 16-18wks	Gait speed in single and dual-task walking	MRI: brain volume, ROI: hippocampus, frontal lobe, and cerebellum
Nagamatsu [61]	HO-AT HO-BT	54(14) 47(29)	67 ± 6 65 ± 6	AT: walking BT: balance and toning exercises	40 min, 1/wk, 52wks	TUG	MRI: brain volume, ROI: basal ganglia
Nieman [79]	HO-AT HO-BT HO-CON	17(5) 19(6) 13(6)	68 ± 3 70 ± 5 69 ± 3	AT: nordic walking BT: mix of balance exercises CON: stretching and relaxing	45–60 min, 3/wk, 52wks	Unipedal stance time EO	MRI: brain volume, ROI: hippocampus
*Studies examining the effects of balance training on neurochemical blood markers of neural adaptation in healthy young individuals*							
N.A							
*Studies examining the effects of balance training on neurochemical blood markers of neural adaptation in healthy older individuals*							
Solianik [80]	HO-BT HO-CON	15(13) 15(13)	60–78	BT: Tai Chi CON: no intervention	60 min, 2/wk, 10wks	CoP velocity during bipedal stance with EO and EC during ST and DT	BDNF
Cekanauskaite [81]	HO-BT HO-CON	18(1) 15(2)	67 ± 6	BT: Yoga CON: no intervention	90 min, 2/wk, 10wks	CoP velocity during bipedal and tandem stance with EO and EC during ST and DT	BDNF
Kubica [34]	HO-BT 1 HO-BT 2 HO-CON	28(13) 15(9) 23(14)	66 ± 1 65 ± 1 64 ± 1	BT: mix of balance exercises BT: Wii Fit Balance board CON: no intervention	30/60 min, 3/wk, 12wks	POMA	BDNF
Szymura [62]	HO. BT PD. BT HO.CON PD.CON	16 (11) 13(8) 16(10) 16(10)	66 ± 3 65 ± 7 67 ± 7 66 ± 4	BT: Uni- and bipedal stance on different devices CON: no intervention	30–60 min, 3/wk, 12wks	POMA	BDNF

*HY* Healthy young individuals, *H* healthy individuals, *HO* healthy older individuals, *PD* Parkinson’s disease patients, *CD* patients with cerebellar degeneration, *BT* balance training, *CON* control, *ST* strength training, *AT* aerobic training, *VR* virtual reality, *BBS* Berg Balance Scale, *TUG* timed up and go, *Hslope and MEPslope* Slope of input–output relation H-reflex and MEP, *MI* motor imagery, *AO* action observation, *AO* + *MI* Action observation together with motor imagery, *Hmax/Mmax* Maximal H-reflex to maximal M-wave ratio

#### Type of Balance Training

Table 1 summarizes the balance interventions used in the included studies. In 19 studies, participants practiced quiet unipedal or bipedal standing on various surfaces such as foam pads, tilt boards, air-filled cushions, and spinning top balance boards. Other balance training protocols comprised: learning to balance on a single unstable device (*n* = 8 studies), exergaming (*n* = 4), Tai Chi (*n* = 5), dancing (*n* = 2), slackline standing and slackline walking (*n* = 3), alpine skiing, which consists of a mixture of strength and balance training (*n* = 1), beam walking (*n* = 1), controlled shifting of the center of gravity (*n* = 1), treadmill slip perturbation training (*n* = 1), and Yoga (*n* = 1).

#### Measures of balance performance

Static balance performance was assessed during bipedal, tandem, and unipedal standing on a rigid surface, moving platform, tilt board, slackline, or spinning top with eyes open or closed, using postural sway outcomes such as the center of pressure (CoP) displacement, CoP area and velocity or outcomes such as the Berg Balance Scale (BBS), an error score or time in balance (*n* = 31 studies). Dynamic balance was assessed by Performance-Oriented Mobility Assessment (POMA), Community Balance Mobility Scale, Timed Up and Go (TUG), perturbed and unperturbed single-task and dual-task treadmill walking, walking negotiating obstacle tasks and beam- and slackline-walking (*n* = 12 studies).

#### Measures of Neural Adaptations

Table 1 shows that 14 of the 42 studies examined the soleus H-reflex following balance training in healthy young individuals (*n* = 9 studies, *n* = 144 participants, 77 males, age: 25 ± 3 years), and healthy older individuals (*n* = 5 studies, *n* = 78 participants, 38 males, age: 70 ± 4 years). The soleus H-reflex was assessed at rest, while sitting, performing plantar flexion, resisting perturbations, and standing on stable and unstable surfaces or platforms.

Adaptations in corticospinal excitability following balance training were assessed in healthy young (*n* = 7 studies, *n* = 85 participants, 49 males, age: 24 ± 2 years) and in healthy older individuals (*n* = 3 studies, *n* = 31 participants, 14 males, age: 67 ± 9 years) using transcranial magnetic stimulation (TMS). The amplitude of TMS generated motor-evoked potentials (MEP), short intracortical inhibition (SICI), long intracortical inhibition (LICI), intracortical facilitation (ICF), the conditioned H-reflex and the slope of the MEP recruitment curve in the soleus, the tibialis anterior and the quadriceps femoris were assessed as measures of corticospinal excitability.

Adaptations in cortical activity were assessed using electroencephalography (EEG, in young individuals 2 studies, *n* = 25 participants, 16 males, age: 24 ± 4 years; in older individuals 1 study), functional magnetic resonance imaging, fMRI (in young individuals 4 studies, *n* = 60 participants, 26 males, age: 25 ± 3 years; in older individuals 3 studies, *n* = 72 participants, 37 males, age: 66 ± 3 years) and functional near-infrared spectroscopy, fNIRS (in younger individuals 0 studies; in older individuals 2 studies, *n* = 45 older individuals, 18 males, age: 72 ± 5 years). EEG data were collected at rest, while performing a beam walking task and in response to balance platform perturbations. fMRI assessed cortical activity during motor imagery, action observation, or in a combination of motor imagery and action observation and at rest to assess functional connectivity. fNIRS was used to assess prefrontal cortical activation during gait.

Structural brain adaptations following balance training were assessed in healthy young (5 studies, *n* = 96 participants, 41 males, age: 32 ± 6 years) and healthy older individuals (6 studies, *n* = 130 participants, 70 males, age: 66 ± 6 years) at rest with eyes closed by MRI to quantify changes in brain volume, cortical thickness, gray matter density, and structural connectivity. Six of these eleven studies examined a specific region of interest, including the hippocampus, basal ganglia, and cerebellum, as well as the parieto-occipital and frontal areas. The five remaining studies did not specify a region of interest but examined whole brain morphological and functional connectivity adaptations to balance training. In addition, four studies examined neural adaptation by assessing blood neurochemicals, i.e., brain-derived neurotrophic factor (BDNF).

### Effects of Balance Training on Balance Performance

The current review focused on the neural correlates of balance learning. We thus performed meta-analyses on balance outcomes following balance training only to characterize the sample of the included studies. Such data have been previously reported in the literature [6, 44]. In the sample of studies included in this review, balance training improved balance outcomes (*g* = 0.96, 95% CI 0.65–1.28, *p* < 0.0001, *I*^2^ = 71%, *n* = 18 studies, k = 60 outcomes, n = 540 participants, Additional file 1: Fig. S2). The omnibus g value of 0.96 includes all changes in balance outcomes produced by acute and chronic balance training in young and older individuals.

The following “Effects of Balance Training on the Trained Balance Task: Skill Acquisition” to “Effects of Balance Training on Balance Skill Retention” sections, comprise the non-meta-analytical systematic synthesis of the changes in balance performance after balance training. “Effects of Balance Training on the Neural Correlates of Balance Learning” section reports on the meta-analytical results for neural correlates of balance training. “Electrophysiological Methods: Spinal Excitability” to “Neurochemical Blood Markers of Neural Adaptation” sections report on the non-meta-analytical systematic synthesis of the neural correlates of balance learning. “Correlated Adaptations in Balance Performance and Neural Adaptation” and “Meta-analytical Associations of Changes in Neural Adaptation with Changes in Balance Performance” sections report on the association between behavior and neural measures.

#### Effects of Balance Training on the Trained Balance Task: Skill Acquisition

**Fig. 2:**
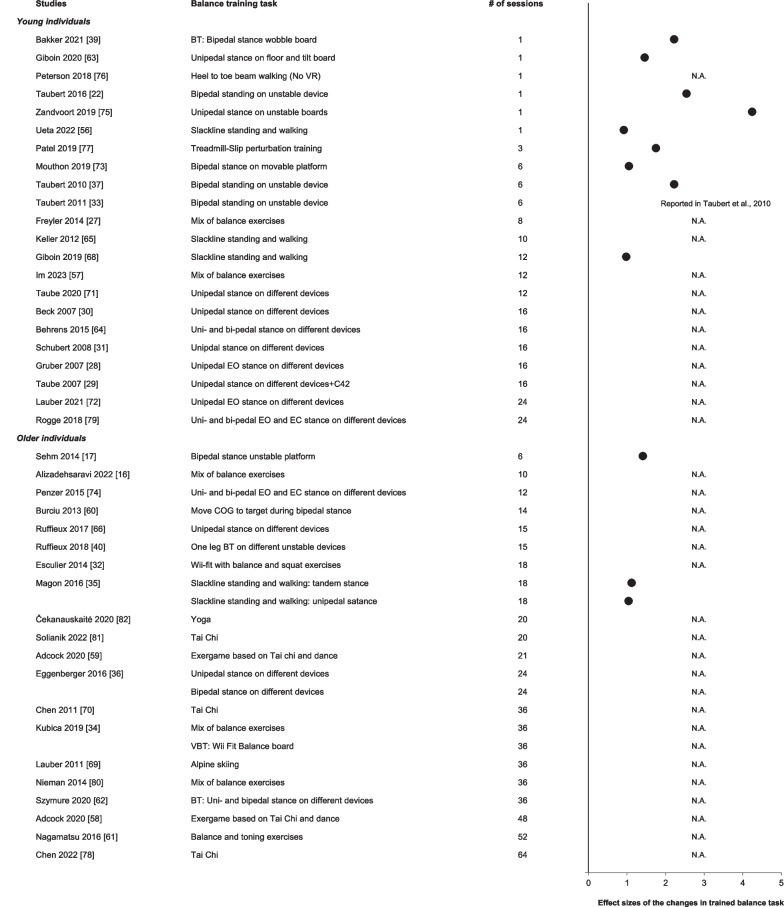
Effect sizes of the changes in the performance of the trained balance task ordered by age group and number of training sessions. Filled symbols: *p* < 0.05. N.A., not applicable: there were no data reported for the improvements in the performance of the trained balance task. These data are shown for sake of orientation and not meta-analyzed. Note how the increase in the number of balance training sessions is not accompanied by a dose-dependent increase in the effect sizes

Figure 2 shows the effect sizes of improvements in the trained balance task, i.e., the magnitude of balance skill acquisition in young (*n* = 9 studies, *n* = 150 participants, 74 males, age: 25 ± 3 years) and older individuals (*n* = 2, *n* = 30, 17, age: 63 ± 4 years). In young individuals, improvements in the trained balance tasks following acute balance training (i.e., within 1 to 3 balance training sessions) ranged from 17 to 207% (*d*:0.9–4.2). Following chronic balance training in young individuals, improvements in the trained balance task ranged from 84 to 117% (*d*:0.9–2.2). No study examined acute balance skill acquisition in older individuals, but after chronic balance training, balance skills in the trained balance task improved from 55 to 478% (*d*:1.0–1.4).

#### Effects of Balance Training on Non-trained Balance Tasks: Skill Transfer to a Static Balance Task

**Fig. 3:**
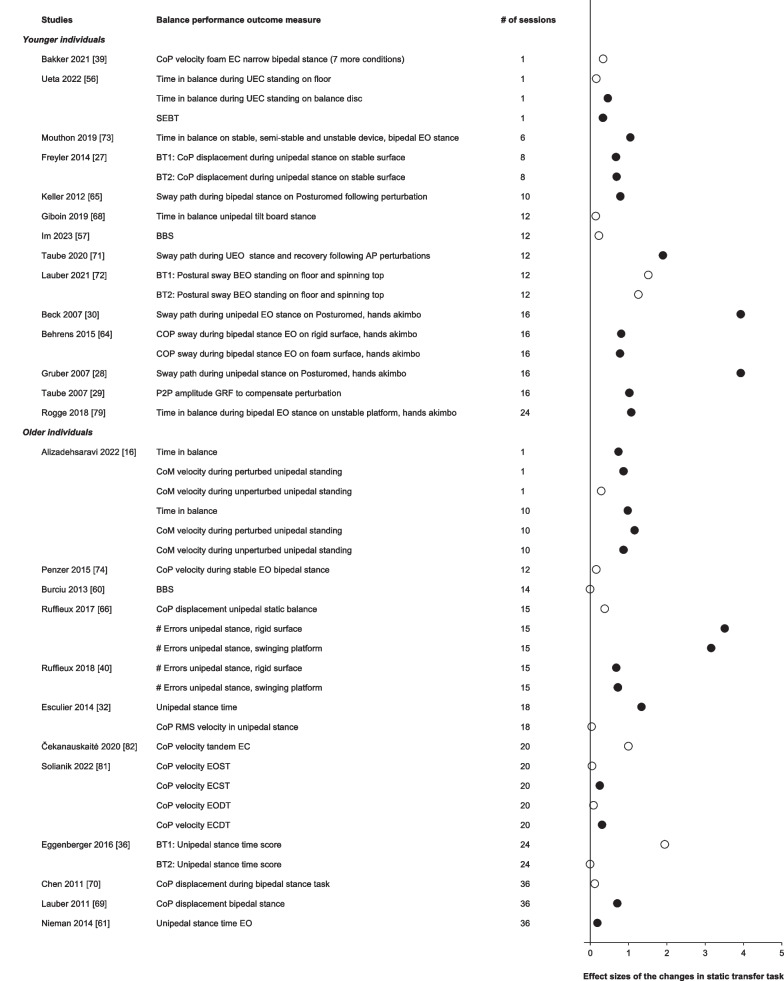
Effect sizes of the changes in the performance of the static balance transfer task ordered by age group and number of training sessions. Open symbols: *p* > 0.05; Filled symbols: *p* < 0.05. These data are shown for sake of orientation and not meta-analyzed. Note how the increase in the number of balance training sessions is not accompanied by a dose-dependent increase in the effect sizes

Figure 3 shows the effects of acute and chronic balance training on static balance tasks that were not trained. Non-trained static balance tasks were assessed in young (*n* = 14 studies, *n* = 206 participants, 109 males, age: 26 ± 3 years) and in older individuals (*n* = 12 studies, *n* = 205 participants, 85 males, age: 69 ± 7 years). In young individuals, two studies reported the effects of acute balance training on the performance of a non-trained static balance task. One of these studies reported a significant increase in unipedal stance time (+ 4%) but only while standing on an unstable balance disk and not while standing on a stable surface [56]. No reductions in the CoP velocity were reported following one day of balance training on an unstable device [39]. Following chronic balance training, the performance of the static balance transfer task improved in 10 out of 12 studies in young individuals by 8% to 84% (*d*:0.7–3.9).

In older individuals, acute balance training (*n* = 1) improved balance performance in a non-trained static transfer task by 18 to 33% (*d* = 0.74–0.87, *p* < 0.05, Fig. 3). Twelve studies reported the effects of chronic balance training on non-trained, static balance tasks in older individuals. Six of these studies reported no improvements in CoP velocity or displacement during bipedal, unipedal, or tandem tasks, BBS scores, or unipedal stance time scores following 12–36 balance training sessions (all *p* > 0.05). In contrast, six other studies reported improvements in the static balance transfer task performance following chronic balance training ranging from 13 to 106% (*d*:0.2–3.5).

#### Effects of Balance Training on Non-trained Balance Tasks: Skill Transfer to a Dynamic Balance Task

**Fig. 4:**
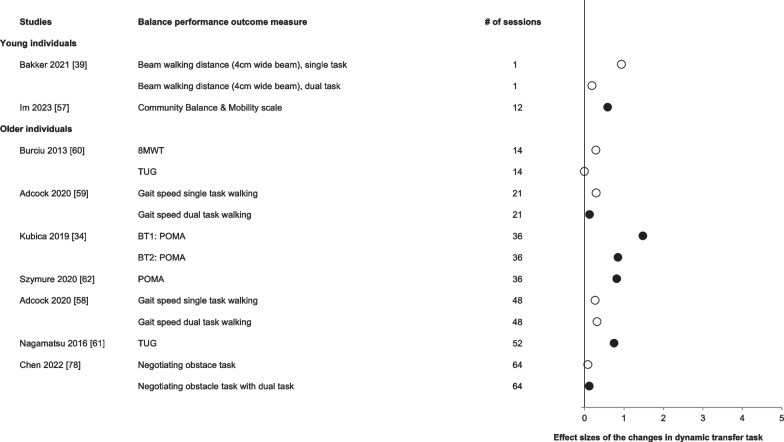
Effect sizes of the changes in the performance of the dynamic balance transfer task ordered by age group and number of training sessions. Open symbols: *p* > 0.05. Filled symbols: *p* < 0.05. These data are shown for sake of orientation and not meta-analyzed. Note how the increase in the number of balance training sessions is not accompanied by a dose-dependent increase in the effect sizes

Figure 4 shows the effects of balance training on dynamic balance skill that was not trained. In young individuals following acute balance training (*n* = 1), no improvements were found in dynamic balance performance. In older individuals, the effects of acute balance training on dynamic balance task skill transfer have not yet been examined.

In young individuals, one study examined the effects of chronic balance training on a dynamic balance transfer task [57]. Twelve weeks of Tai Chi practice resulted in an increased performance on the Community Balance & Mobility Scale. In older individuals, 7 studies (*n* = 171 participants, 95 males, age: 67 ± 5 years) examined the effects of chronic balance training on skill transfer to a dynamic balance task and reported mixed results. Six weeks of Tai Chi and dance-based exergaming balance training significantly increased gait speed during dual-task but not single-task walking [58]. However, another study performing the same intervention for 48 sessions over 16–18 weeks of training did not increase gait speed in either single-task or dual-task walking (*p* > 0.05) [59]. In addition, daily balance training over 2 weeks did not improve performance on the 8-m walk test or the TUG score (*p* > 0.05) [60]. However, TUG scores did improve after a year of weekly balance training [60, 61]. Similarly, after 12 weeks of a Wii-fit exergame and balance training consisting of a mixture of balance tasks, POMA scores improved [34, 62].

#### Effects of Balance Training on Balance Skill Retention

Four studies reported on the retention of the acquired balance skill following a no-intervention period of 1 week [39], 2 weeks [16], 3 months [37] or 20 months [17]. While these studies reported a decline in balance performance at retention compared to posttest, balance performance improved persistently at retention compared to performance at baseline. Even 20 months post-intervention, a pre-retention increase of 23% was reported (*d*: 2.36) [17]. One study reported the retention of a non-trained (transfer) balance task [60].

### Effects of Balance Training on the Neural Correlates of Balance Learning

**Fig. 5:**
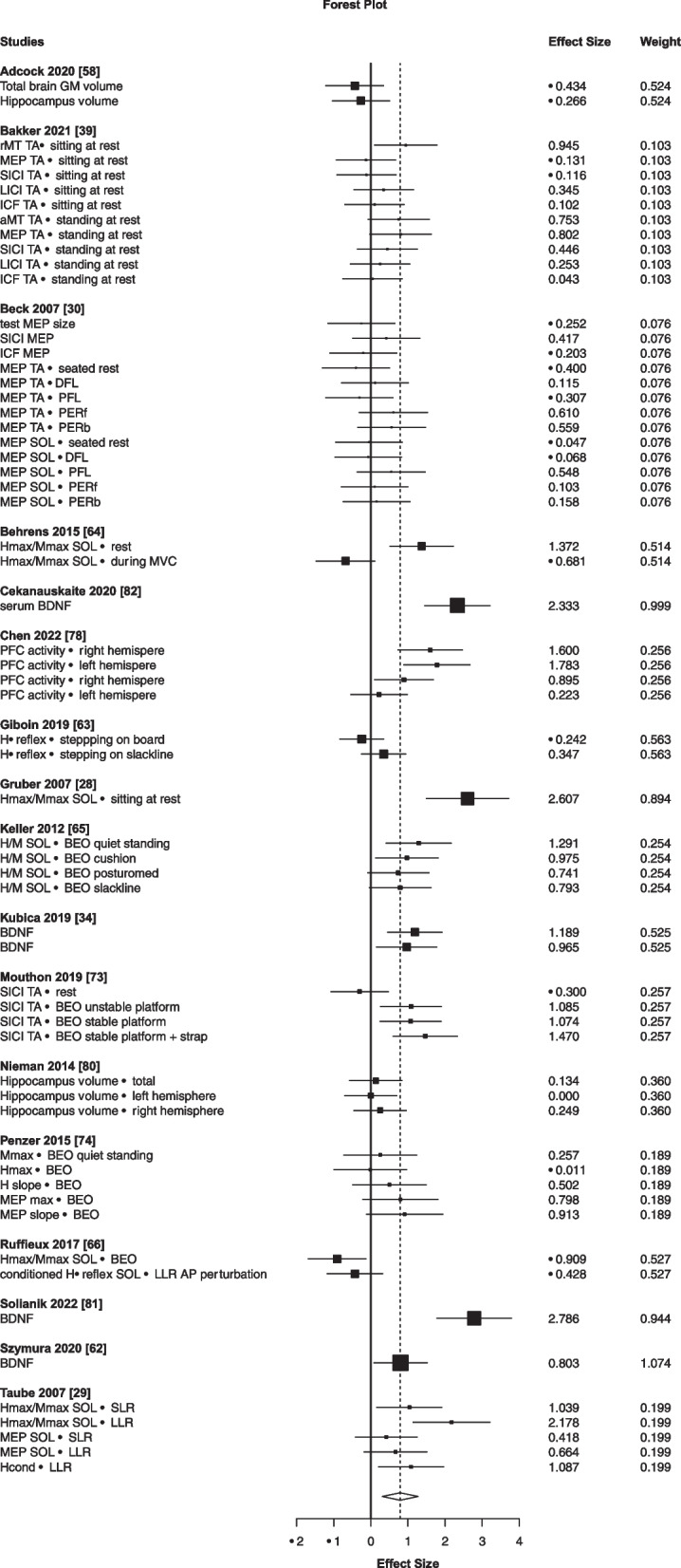
Forest plot showing the effects of balance training interventions on neural adaptations in 508 participants, 17 studies and 62 outcomes (the open diamond symbol reflects the pooled effect size: *g* = 0.79, 95% CI 0.306–1.278, *p* < 0.01, *I*^2^ = 82%). Positive effect sizes denote favorable effects of balance training on neural adaptation outcomes. *GM* Gray matter, *rMT* Resting motor threshold, *MEP* motor-evoked potential, *SICI* Short intracortical inhibition, *LICI* Long intracortical inhibition, *ICF* Intracortical facilitation, *TA* Tibialis anterior, *SOL* Soleus, *DFL* Dorsal flexion, *PFL* Plantarflexion, *PERf* Perturbation forward, *PERb* Perturbation backwards, *BDNF* Brain-derived neurotrophic factor

Because the meta-analysis of the balance performance in the sample of studies included in this systematic review indicated that balance training improved balance performance outcomes, it was meaningful to further explore the neural correlates of balance training-induced improvements in balance performance. Figure 5 shows that the effect size of the omnibus analysis examining the effects of balance training on neural adaptation was high (*g* = 0.79, 95% CI 0.306–1.278, *p* < 0.01, *I*^2^ = 82%, Table 2). This omnibus effect size value of g = 0.79 comprises all acute and chronic interventions in both age groups pooled. Subgroup analyses (Table 2) were significant for young (*g* = 0.74, 95% CI 0.11–1.38, *p* < 0.05, *I* = 78%) but not healthy older individuals (*g* = 0.83, 95% CI − 0.03 to 1.70, *p* > 0.05, *I* = 86%). Subgroup analysis (Table 2) for acute interventions had insufficient study numbers (≤ 4), meaning its results are unreliable. Subgroup analysis for chronic interventions produced a significant effect (*g* = 0.82, 95% CI 0.30–1.34, *p* > 0.05, *I* = 83%). In addition, Additional file 1: Table S1 shows that none of the outcome measures (training duration, population) significantly moderated the effects of balance training on the neural correlates of balance learning. We, therefore, performed a systematic synthesis of the neural correlates of balance learning and present these in “Electrophysiological Methods: Spinal Excitability” to “Neurochemical Blood Markers of Neural Adaptation” sections.

**Table 2:** Overall and subgroup analyses for the effects of balance training on neural adaptations

Variable	Effect size		95% CI			Heterogeneity		Frequencies		
	*g*	SE	LL	UL	*p*	*I*^2^	Tau^2^	*k*	*N*_s_	*N*_p_
All	0.79	0.23	0.31	1.28	0.003	82	0.80	62	17	508
*Population*										
Healthy young	0.74	0.27	0.11	1.38	0.028	78	0.64	41	8	216
Healthy older	0.83	0.38	− 0.03	1.70	0.057	86	1.05	21	9	292
*Training duration*										
Acute	**0.34**	**0.00**	**0.344**	**0.34**	**0.000**	**77**	**0.00**	**10**	**1**	**24**
Chronic	0.82	0.24	0.304	1.34	0.004	83	0.00	52	16	484
*Neural adaptation outcome measure*										
Spinal	0.66	0.42	− 0.37	1.69	0.167	86	1.15	15	7	191
Corticospinal	0.38	0.22	− 0.18	0.94	0.138	56	0.25	33	6	149
Structural brain	** − 0.09**	**0.24**	** − 3.11**	**2.93**	**0.773**	**0**	**0.00**	**5**	**2**	**63**
Neurochemical	**1.71**	**0.48**	**0.19**	**3.22**	**0.037**	**79**	**0.69**	**5**	**4**	**158**

Bold-highlighted sections indicate unreliable subgroup analyses due to a low number of studies

*g* Effect size, *SE* Standard error of the effect size, *CI, LL, UL* 95% Confidence interval of the effect size and its lower and upper limit, *p* Probability, *I*^2^ Proportion of total variability due to between-study heterogeneity, *Tau*^2^ Between-study variance, *k* Number of outcomes, *N*_s_ Number of studies, *N*_p_ Number of participants

#### Electrophysiological Methods: Spinal Excitability

**Fig. 6:**
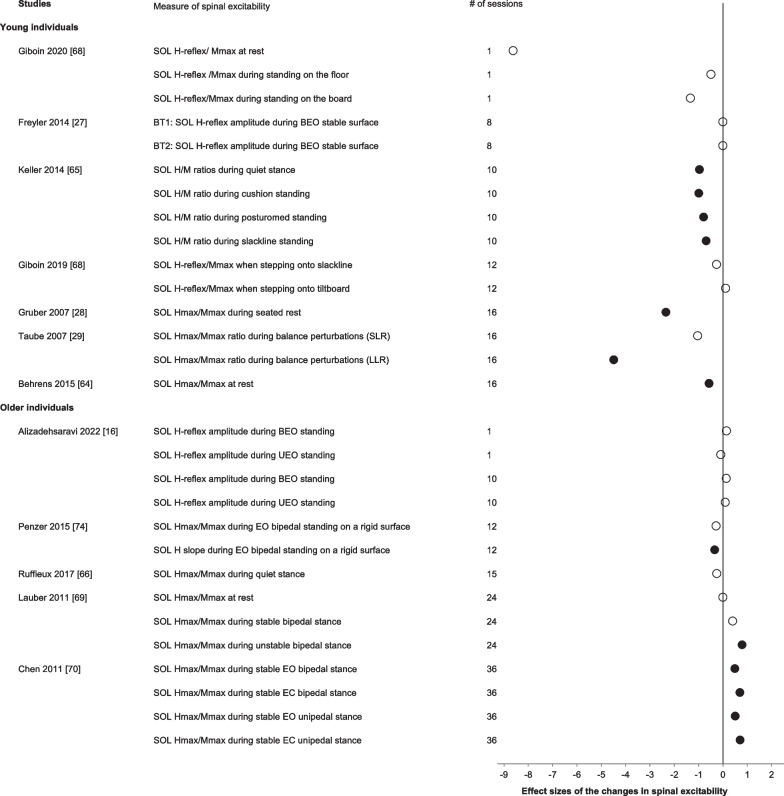
Effect sizes of the changes in measures of spinal excitability ordered by age group and number of training sessions. Open symbols: *p* > 0.05. Filled symbols: *p* < 0.05. N.A. indicates data necessary to calculate effect sizes were not available. The subgroup analysis was not significant for the spinal excitability measures and only included 7 studies; we therefore performed a systematic synthesis of the adaptations in spinal excitability in response to balance training in all available studies. Note how the increase in the number of balance training sessions is not accompanied by a dose-dependent increase in the effect sizes

The subgroup analysis for spinal excitability comprised all acute and chronic balance training interventions examining spinal measures in the two age groups and was not significant (*g* = 0.66, Table 2). We, therefore, performed a systematic synthesis of the adaptations in spinal excitability in response to balance training. Figure 6 summarizes the soleus H-reflex data in young (*n* = 7 studies, *n* = 121 participants, 63 males, age: 25 ± 3 years) and older individuals (*n* = 5, *n* = 66 participants, 32 males, age: 71 ± 5 years). Acute [63] and chronic balance training in young individuals [28, 64] resulted in reductions in the soleus H-reflex amplitude when assessed during seated rest (− 18 to − 34%; *d*:0.57–8.62). Chronic balance training in older individuals consisted of 29 skiing sessions, a mixture of balance and eccentric strength training, and did not modify soleus H-reflex assessed during seated rest.

When the soleus H-reflex was assessed during quiet upright standing following chronic balance training in young individuals, 4 weeks of balance training consisting of a mixture of balance tasks did not modify the soleus H-reflex [27]. In contrast, 10 sessions of slackline training reduced the soleus Hmax/Mmax ratio by 24% when balancing on the slackline [65]. In older individuals, one balance training session did not result in adaptations of the soleus H-reflex [16]. Also, chronic balance training consisting of a mixture of unipedal balance tasks over three to 5 weeks did not modify soleus H-reflex and assessed during quiet upright standing [16, 66]. Excitability was reduced when the soleus H-reflex was assessed during a balancing task after 6 weeks of combined balance and strength training. However, this adaptation was detected with the slope of the stimulation intensity and H-reflex amplitude relationship (− 39%) [67].

Eight studies examined adaptations in H-reflex while standing on an unstable board or surface or while responding to perturbations. In young individuals, significant reductions in H-reflex amplitudes during the performance of these tasks occurred following chronic balance training [29–31, 65, 68]. In contrast, 12 weeks of alpine skiing [69] or Tai Chi training [70] increased soleus H-reflex excitability in older individuals (Fig. 6).

#### Electrophysiological Methods: Corticospinal Excitability

**Fig. 7:**
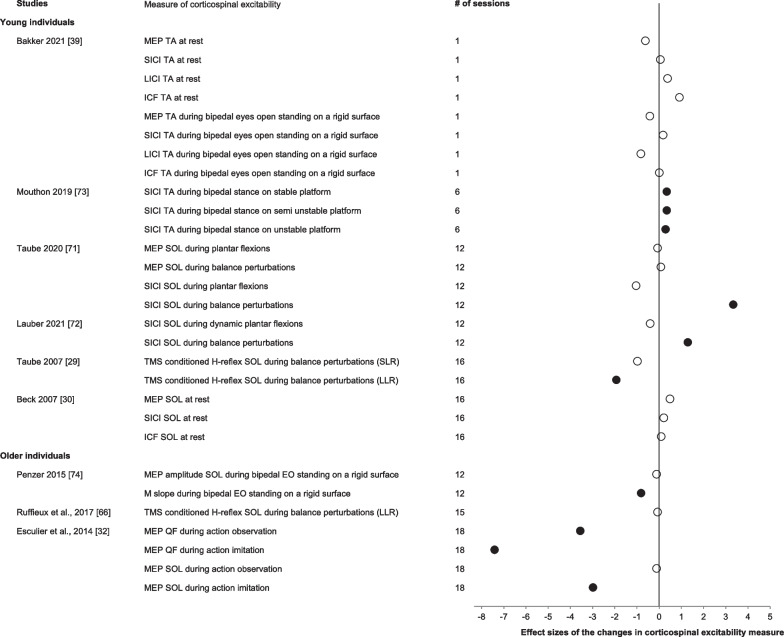
Effect sizes of the changes in measures of corticospinal excitability in young and older individuals ordered by age group and number of training sessions. Open symbols: *p* > 0.05. Filled symbols: *p* < 0.05. N.A. indicates data necessary to calculate effect sizes were not available. The subgroup analysis was not significant for the corticospinal excitability measures and only included 6 studies; we therefore performed a systematic synthesis of the adaptations in corticospinal excitability in response to balance training in all available studies. Note how the increase in the number of balance training sessions is accompanied by inconsistent changes in the effect sizes

The subgroup analysis for corticospinal excitability comprised all acute and chronic balance training interventions examining corticospinal measures in the two age groups and was not significant (*g* = 0.38, Table 2). We, therefore, performed a systematic synthesis of the adaptations in corticospinal excitability in response to balance training. Figure 7 summarizes the corticospinal excitability data. The neural correlates of balance training were examined using TMS in young (*n* = 7 studies, 85 participants, 49 males, age: 24 ± 2 years) and older individuals (*n* = 3, *n* = 31 participants, 14 males, age: 67 ± 9 years). MEPs, SICI, LICI, and ICF, as well as the TMS-conditioned H-reflex, were assessed over the motor cortex area of the tibialis anterior, soleus, and quadriceps femoris muscles. In young individuals, acute balance training consisting of one session on an unstable board did not modify MEP, SICI, LICI, or ICF when assessed at rest (change: 4–75%, *d*:0.05–0.91) or during bipedal quiet standing (change:1–66%, *d*:0.01–0.82) [39]. The effects of acute balance training on corticospinal excitability have not yet been assessed in older individuals.

Assessing the effects of chronic balance training on corticospinal excitability in young individuals at rest produced no changes in soleus MEP, SICI, or ICF (change:7 to 12%, *d*:0.09 to 0.49) following 4 weeks (16 sessions) of unipedal stance balance training on different devices [30]. Four weeks (12–16 sessions) of unipedal balance training on multiple devices also did not modify the conditioned H-reflex (TMS over the cortical representation of the soleus) when assessed during the spinally generated short latency response (− 5%, *d*:0.98) or during balance perturbations (− 3%, *d*:0.07). Similarly, no effects were observed in a non-trained control task (i.e., plantarflexions, + 3%, *d*:0.08). However, conditioned H-reflexes elicited at the cortically mediated long latency response after balance perturbations were significantly reduced (− 44%) in response to balance training [29]. Also, SICI measured during non-trained plantar flexion did not significantly change (− 50 to − 20%, *d*:0.4 to 1.04), but SICI significantly increased when measured during balance perturbations (+ 124 to 256%, *d*:1.29 to 3.34) following 4 weeks (12 sessions) of unipedal stance balance training on multiple devices [71, 72]. In addition, following 2 weeks (6 sessions) of balance training on a movable platform, SICI increased when measured on the balance platform but remained unchanged when measured at rest (stable platform: 48–51%, *d*:0.29–0.35; semi-unstable platform: 52%, *d*:0.35) [66, 73].

Examining the effect of chronic balance training on the corticospinal excitability in older individuals showed that 5 weeks of balance training consisting of a mixture of balance tasks did not modify the conditioned soleus H-reflex (− 5%, *d*:0.07) assessed during bipedal standing while experiencing rapid backward translations [66]. While a 6-week balance training consisting of a mixture of balance tasks also did not modify MEP size (− 5%, *d*: 0.12), the slope of the recruitment curve, assessed during eyes open bipedal standing on a rigid surface, decreased significantly (− 44%, *d*:0.81) [67]. In addition, significant reductions in MEP amplitude of the quadriceps femoris took place during both action observation (− 29%, *d*:3.55) and imitation (− 14%, *d*:7.41) as well as in the soleus during action imitation (− 11%, *d*:2.98) of a mini-squat action following 6-weeks (18 sessions) of Wii-fit balance training [32].

#### Imaging Methods: Cortical Activity

Only one study examining cortical activity outcomes was included in the meta-analysis. We, therefore, performed a systematic synthesis of the adaptations in cortical activity in response to balance training. Twelve studies examined the effects of balance training on cortical activity, using EEG during beam walking and at rest, fMRI during motor imagery (MI), action observation (AO), a combination of AO + MI, fMRI at rest examining functional connectivity or by fNIRS during walking. In young individuals, adaptations in cortical activity following acute balance training were assessed in 6 studies (*n* = 85 participants, 42 males, age: 25 ± 3 years). One 30-min session of unipedal stance balance training on unstable boards decreased coherence between relevant muscle synergies and the left paracentral lobule synergy in the 40-Hz band (Piper rhythm) [74]. A 30-min session of heel-to-toe beam walking increased alpha power in the occipital and parietal areas and decreased power in the gamma band [75] and 30-min of slackline training increased resting-state functional connectivity between the left prefrontal cortex and the medial areas in the primary sensorimotor area [56]. Three consecutive days of treadmill slip perturbation training increased activity in several cortical regions (left middle frontal gyrus, right superior parietal lobule, right inferior occipital gyrus, and left lingual gyrus) during motor imagery of slipping and walking tasks [76]. Also, fronto-parietal network connectivity increased 1 week after two brief dynamic balance training sessions [33]. No studies examined the effects of acute balance training on cortical activity in older individuals.

Two studies examined the effects of chronic balance training on cortical activity in young individuals. Repeated training sessions over six consecutive weeks increased functional connectivity in various cortical and subcortical brain structures [33, 68]. Moreover, Taubert et al. [33] reported that the magnitude of adaptation was in accordance with individual performance improvements. In older individuals, six studies examined cortical activity following 15–64 sessions (*n* = 136 participants, 64 males, age: 69 ± 4 years). Both 15 sessions of unipedal balance training and 36 balance training sessions consisting of a mixture of balance tasks and virtual balance training reduced brain activity during action observation and motor imagery of balance tasks in areas responsible for postural control, e.g., the primary motor cortex, the primary somatosensory cortex, and the left ventral premotor cortex [34, 40]. These data agree with the reduced oxygenation in the right and left hemispheric prefrontal cortex assessed by applying fNIRS during walking following balance training [36]. However, 64 sessions of Tai Chi resulted in an increased PFC activity during the execution of a negotiating obstacle task with an added cognitive task [77]. No functional connectivity changes were reported after 6 weeks of balance training. A sub-analysis of responders, i.e., participants with improved slackline performance after slackline balance training, revealed reduced connectivity between the striatum and other brain regions such as the caudal anterior cingulate gyrus, the precuneus, superior parietal lobe, and the supramarginal gyrus as well as reduced connectivity between the supplementary motor cortex and the supramarginal gyrus [35]. In contrast, 21 sessions of Tai Chi over 7 weeks did not result in adaptations of the cortical activity as assessed by resting-state EEG [59].

#### Structural Brain Outcomes

The subgroup analysis for structural brain outcomes had insufficient study numbers per random variance analysis guidelines (≤ 4). We, therefore, performed a systematic synthesis analysis of the adaptations in structural brain outcomes in response to balance training. Imaging studies examined structural brain adaptations in response to acute and chronic balance training by MRI, investigating gray and white matter volume, structural connectivity, cortical thickness, and gray matter density (*n* = 5 studies in young individuals, *n* = 96 participants, 41 males, age: 32 ± 6 years; *n* = 6 studies in older individuals, *n* = 130 participants, 70 males, age: 66 ± 6 years). In young individuals, acute balance training (i.e., one 45-min session) on an unstable device significantly increased cortical thickness and gray matter density in the primary motor area representing the foot and trunk [22]. In addition, 90 min of practice on a complex balancing task within 2 weeks resulted in macroscopic structural gray matter adaptations in frontal and parietal brain areas [37]. However, 30 min of slackline training did not induce adaptations in gray matter volume or white matter microstructure [56]. The effects of acute balance training on structural brain adaptations in older individuals have not yet been assessed.

Three studies examined the effects of chronic balance training (6–24 training sessions) on unstable devices in younger individuals. Improvements in balance performance were accompanied by strengthened cerebro-cerebellar and interhemispheric structural connections [57], an increased cortical thickness [78], reduced volume of the putamen [78] and increases in gray matter volume [37]. Despite the structural gray matter adaptations being reported following 2 weeks of balance training, these adaptations could no longer be detected after chronic balance training (i.e., 6 weeks).

Three of six studies that examined the effects of chronic balance training reported no changes in brain volume in older individuals (*p* > 0.05). Also, no adaptations in brain volume were reported following 52 exergame balance training sessions [58] or 18 sessions of slackline training [35]. Following a 1-year balance training period, older individuals showed no changes in left putamen volume while mobility improved [61]. However, older individuals who declined in mobility level had a decreased left putamen volume (*p* < 0.05), indicating that balance training may contribute to the maintenance of brain volume in key regions responsible for motor control [61]. In addition, three other studies reported structural adaptations following chronic balance training in older individuals (i.e., 6–156 sessions). Six weeks of bipedal balance training on a moveable platform reduced right hemisphere cerebellar gray matter volume (lobules V-VI) [17] and 14 balance training sessions over 2 weeks and 156 balance training sessions over 1 year increased gray matter volume in older individuals in the cerebellum [60] and hippocampus [79].

#### Neurochemical Blood Markers of Neural Adaptation

The subgroup analysis for neurochemical blood markers of neural adaptation had insufficient study numbers per random variance analysis guidelines (≤ 4). We, therefore, performed a systematic synthesis of the adaptations in neurochemical blood markers in response to balance training. Neurochemical blood markers of neural adaptation were only examined following chronic balance training in older individuals (*n* = 4 studies, *n* = 92 participants, 37 males, age: 67 ± 3 years. BDNF increased by 66–101% (*d*:0.39–1.51) after 20–36 balance training sessions consisting of a mixture of balance tasks [34, 62], Tai Chi [80] or Yoga balance training sessions [81] over 10–12 weeks, but not after 36 Wii-fit balance board balance training sessions over 12 weeks [34].

### Correlated Adaptations in Balance Performance and Neural Adaptation

In young individuals, a positive correlation was reported between gray matter volume adaptations in frontal and parietal areas and improvements in balance performance following two balance training sessions consisting of standing on an unstable device [37]. However, time in balance on an unstable board did not correlate with changes in MEP, SICI, and LICI when assessed at rest following a single balance training session [39]. Six balance training sessions on an unstable platform resulted in a correlated increase in SICI during the execution of a balance task (*r*:0.53–0.56) [73]. Another study consisting of 16 balance training sessions over 4 weeks reported that changes in the TMS-conditioned H-reflex (i.e., changes in the excitability of the fastest corticospinal pathways) were related to improvements in stance stability (*r* = 0.87) [43]. In addition, both an increase in cortical thickness in the left precentral gyrus (*r* = 0.66) and a decrease in left putamen volume (*r* = − 0.48) correlated with an increase in balance performance following 24 balance training sessions over 12 weeks [78]. Following both a single and 10 balance training sessions, no correlation between H-reflex gains and balance improvements was reported in older individuals [16]. However, 6 weeks of balance training on a moveable platform indicated positive correlations between individual balance improvements and gray matter volume changes, but only in the left hippocampus [17]. A 10-week Yoga program also led to correlated improvements in balance performance and changes in BDNF (*r*:0.41–0.71) [81]. Other studies did not compute or report the associations between the improvements in balance performance and neural adaptation.

### Meta-analytical Associations of Changes in Neural Adaptation with Changes in Balance Performance

The association between effect sizes reflecting changes in neural adaptation and the effect sizes reflecting changes in balance performance was not significant (*n* = 7 studies, *p* = 0.995). The association between effect sizes reflecting changes in neural adaptation assessed during a transfer task and the effect sizes reflecting changes in transfer balance performance was also not significant (*n* = 6, *p* = 0.660).

### Assessment of Methodological Quality

Additional file 1: Table S2 shows that the quality of all studies included in this review ranged between 1 and 6, with a mean of 5 and a median of 6. All studies included in the meta-analysis scored 6 on the PEDro score indicating ‘good’ methodological quality*.*

### Risk of Bias and Sensitivity Analyses

A visual inspection of the funnel plot complemented with Egger’s regression tests for funnel plot asymmetry indicated potential publication bias resulting in a possible overestimation of the summary effect on neural adaptation (*z* = 3.53, *p* < 0.001, Additional file 1: Fig. 3).

Sensitivity analyses of the neural adaptation outcomes revealed that removing the one identified influential case did not affect the results (Δ*g* =  − 0.12, SE = 0.31, *p* = 0.699; Additional file 1: Fig. S4).

## Discussion

We systematically reviewed the effects of age on the neural correlates of balance skill learning measured during the performance of static (standing) and dynamic (walking) balance tasks. Meta-analysis revealed that balance training had an overall large effect on the neural correlates of balance skill learning (*g* = 0.79, Fig. 5, Table 2). Neither age nor training duration moderated these improvements. Systematic synthesis focused on the effects of acute (≤ 3 sessions) and chronic balance training (i.e., > 3 sessions) on the neural correlates of balance skill acquisition, transfer (specificity), and retention. We found that young and older individuals improved balance performance rapidly within 1–3 training sessions. However, the acute balance training-induced gains in balance skills improved little with additional sessions (Figs. 2, 3, 4), i.e., with chronic balance training. Improvements in balance performance occurred mainly in the trained and less in the non-trained balance (i.e., transfer) tasks. Our systematic synthesis also seems to suggest little correspondence (associations) between improvements in balance skills and changes in measures of spinal, cortical, and corticospinal excitability in young individuals and older individuals and between the time courses of these changes up to 64 sessions. We discuss these findings with a perspective on the magnitude, rate, and specificity of balance skill learning and its neural correlates in the two age groups.

### Adaptations in Balance Performance

In line with previous reviews examining the effect of balance skill training on balance skill performance [6, 44], our meta-analysis, performed for the sake of orientation only, revealed positive effects of balance training on measures of balance performance (*g* = 0.96, Additional file 1: Fig. S2). The current review focused on the neural correlates of balance learning; we, therefore, discuss only non-meta-analytical findings regarding the effect of balance training on balance performance.

#### Adaptations in Balance Skill Performance of the Trained Task

Increasing evidence suggests that akin to acquiring manual motor skills, where rapid improvements occur within a single training session [14, 82], only a few minutes of balance training strongly improves balance performance in the trained task (Fig. 2). Such improvements are expected and are in line with the conclusions of a previous review [18]. When the training task is also the test task, improvements are large [18]. We extend the results of this previous review by including more studies in young individuals, adding data in older individuals, and by organizing the data according to training dose, i.e., number of sessions (Fig. 2, *n* = 10 studies altogether).

Our review revealed that chronic balance training did not further increase gains in trained task balance performance produced by acute balance training. Indeed, one balance training session on unstable boards improved standing balance on those boards with an effect size of ~ 2 times greater than 3–6 sessions of balance training (Fig. 2). It seems that improvements in the trained balance task would tend not to improve beyond what the improvements were already after 30 min. No study has yet examined the effects of acute balance training on balance performance of the trained task in older individuals. However, 18 sessions of slackline training improved balance performance similar to those achieved after just a few sessions, regardless of age [35]. The data suggest that balance skill acquisition can occur in minutes after practicing a great variety of tasks and that these rapid initial improvements tend not to improve further after even up to 17 additional exercise sessions (Fig. 2). It remains to be seen if a relative reduction in the balance stimulus over sessions due to learning of the balance skill or other factors account for lack of balance training dose effect on balance performance in the trained task. Repetitive balance practice could still be necessary to retain the trained balance skill. However, due to a lack of systematic examination of retention of previously practiced balance skills, we are not yet able to determine if the number of training sessions is associated with the magnitude of balance skill retention: only 5 studies reported retention of the trained or non-trained balance skill following a no-intervention period of 1 week up to 20 months [16, 17, 39, 60, 63].

#### Adaptations in Balance Performance of the Non-trained (Transfer) Task

The effect sizes reflecting improvements in the non-trained (transfer) balance tasks were about one-half of the effect sizes reflecting improvements in the trained task (Figs. 3, 4, 5). These changes did not improve further with additional balance training sessions, a phenomenon we discussed above relative to the trained task. At the extreme, completing 36 or even 52 exercise sessions, static and dynamic transfer tasks revealed no more improvements in balance than the improvements produced by one balance training session. Balance training failed to produce significant changes in 8 out of 24 studies examining a non-trained static transfer task and 3 out of 7 studies examining a dynamic transfer task (Figs. 3,4). Thus, transfer of the learned balance skill to a non-trained balance task is limited or at least challenging. When the spatiotemporal features, the dynamics, or the muscle activation patterns of the training and testing (transfer) tasks differ, there could be limited or even no improvements in the performance of the transfer balance task. These data draw attention to the possibility that one form of balance training would improve balance performance in the trained but not any other balance (transfer) test tasks and that such changes can be expected to occur already after acute balance training. Also, following chronic balance training lasting up to 1 year, performance in the non-trained transfer task would increase only to a limited extent. We extended these training specificity data reported previously [18] to older individuals (Figs. 2, 3, 4). How the task-specificity of balance learning influences the effectiveness of intervention protocols aiming to reduce fall incidence remains to be examined.

### Neural Adaptations After Balance Training

Meta-analysis of the effects of balance training revealed that overall, balance training improves neural measures (Table 2, Fig. 5). However, neither age nor training duration moderated this effect (Additional file 1: Fig. S2). Subgroup analyses examining studies including young and older individuals separately resulted in a significant effect only for young individuals, which might indicate that the measures designed to capture neural adaptations are not sensitive enough in older individuals.

The training specificity of balance training might be related to the training-induced specificity of the neural command and activation of muscles, restricting the generalizability of balance training effects to any non-trained balance skill. This might imply that brain, spinal cord, and muscle activation levels and patterns would be unique to the trained task that would restrict balance improvements in non-trained (transfer) tasks. We, therefore, examined if markers of neural adaptation paralleled the behavioral data, i.e. greater adaptations when measured in the trained than in the non-trained task. Such information could then be broadly interpreted as meaning that neural adaptation would underlie balance training induced training specificity, an idea examined by individual experimental studies [31, 63, 71, 73] but not yet systematically reviewed.

#### Adaptations in Spinal Excitability

In young individuals, H-reflex tended to decrease following balance training, but the between-study variation was high (Fig. 6, upper panel). The effect sizes in 4 of 7 studies or only in 7 of 15 H-reflex outcomes were significant. Perhaps there is a threshold for the number of sessions for H-reflex to decrease because significant decreases started to appear after 16 sessions. Still, the pattern of effect sizes in relation to session number failed to reveal a consistent dose–response relationship. The many non-significant changes require additional studies using consistent outcomes and recording conditions.

Whether changes in H-reflex follow a similar pattern compared with the increases in balance performance is difficult to ascertain in young individuals. One reason is that only 3 of 9 studies assessed the H-reflex in the trained task (Fig. 6, upper panel). These 3 studies revealed an inconsistent pattern. Two of these studies suggested high levels of task-specificity, where adaptations in H-reflex were visible only when assessed during the trained balance task but not during a non-trained (transfer) task [31, 68]. Other studies revealed adaptations in H-reflex also during a non-trained task or even at rest. Further, systematic synthesis revealed that H-reflex outcomes decreased the most, up to 34%, in sitting, which is the least specific posture relative to the trained tasks [28, 30, 64]. For standing, the reductions were 24% [63, 65] and the improvements were least, 20%, when the test task most closely resembled the training task [31, 68]. To increase our understanding of the potential spinal mechanisms of balance training, future studies need to examine changes in balance performance and H-reflexes in the trained and non-trained tasks at baseline, after a few sessions, and after chronic balance training.

The pattern of effect sizes in H-reflex after balance training appeared to differ between young vs. older individuals. The interpretation of data is complicated by a lack of acute balance learning data in the trained task (Fig. 2, lower panel) and the predominantly non-significant changes in the balance transfer task (Figs. 3, 4, lower panels). In older individuals, only one study examined the effects of acute balance training on H-reflex, and these changes were not significant (Fig. 6, lower panel) [16]. The age differences become apparent after chronic balance training: decreases in H-reflex in young individuals contrasted with no changes or increases in H-reflex in older individuals (Fig. 6, lower panel). One interpretation of these data could be related to H-reflex size at baseline: young individuals show up to ~ 50% higher H-reflex size in various postures at baseline compared to older individuals [83]. The increases in H-reflex after balance training in older individuals might reflect adjustments toward the normal-young excitability. However, the data need to be interpreted with caution due to the repeat outcomes from only two studies [69, 84] and because non-significant changes and even decreases in H-reflex after 12–15 sessions can also occur [66, 67]. Penzer et al. [67] also used the slope of the H-reflex recruitment curve as an outcome that measures the excitability of the entire motoneuron pool vs. the most often used measure of peak H-reflex amplitude, focusing on one point on the H-reflex recruitment curve.

Reductions in Ia-transmission via increased presynaptic inhibition seem to be the main reason for reductions in H-reflex after balance training in young individuals regardless of session number (Fig. 5). Presynaptic inhibition controls the excitation of the postsynaptic neuron by the Ia afferents. The postsynaptic neuron can thus still receive (direct) inputs without presynaptic modulation. In essence, the reduction in presynaptic inhibition reduces the contribution of spinal reflexes to balance control and increases the relative role of supraspinal inputs in controlling balance. The functional interpretation is that inhibition of spinal reflexes in balance tasks may reduce joint oscillations with movement control shifted to higher centers [2, 29, 83, 85]. A dysfunction in the presynaptic inhibition circuit, more corticospinal control during balance tasks, or a combination of these factors might be involved in postural control after balance training in older individuals [29, 30, 86].

In summary, our systematic synthesis suggests little temporal correspondence between how balance performance and H-reflex outcomes changed. These changes also revealed a lack of dose effect with respect to the number of sessions. The H-reflex changes perhaps reflect a general training effect rather than a training task-specific effect. This observation is also supported by a lack of association between balance skill changes and H-reflex metrics changes after balance training in studies that reported such associations [16]. That is, it seems at best it is unclear whether changes in H-reflex following balance training underlie the behaviorally observed task-specificity observed after balance training in young and older individuals.

#### Adaptations in Corticospinal Excitability

Following balance training in young individuals, reductions in cortical motor contributions occurred mainly during perturbations or standing on an unstable platform but not at rest [30, 39] and only in one study during normal quiet standing [73]. In older individuals, soleus corticospinal excitability during quiet standing decreased after balance training, but only when excitability was indexed by the slope of the stimulation intensity vs. motor-evoked potential amplitude relationship. The recruitment slope encompasses excitability changes in all of the corticospinal cells the TMS pulse accesses, instead of just a portion of the corticospinal cells captured by the maximal MEP size, which did not change [67].

Reductions in cortical motor contributions were reported in both young and older individuals and were revealed by decreased cortical/corticospinal excitability [29, 32, 67, 71] increased levels of intracortical inhibition [71–73] and increased active motor thresholds during postural task execution [73] (Fig. 7). In line with adaptations following learning of a manual motor skill [87], balance training may lead to a ‘shift in movement control’ from cortical to more subcortical and cerebellar structures [43]. The effect size of the changes in corticospinal excitability does not seem to depend on the number of training sessions, but more on the task during which the corticospinal excitability is assessed. Training effects in young and older individuals were most apparent during the performance of the trained task and were dependent on the training modality. These results suggest that, in line with the changes in balance skill performance, changes in corticospinal excitability are task-specific and independent of age.

#### Adaptation in Cortical Activity as Examined by Neuroimaging

The adaptations in cortical activity reviewed here are mostly in line with previously reported manual skill acquisition studies. These manual skill learning studies show increased cortical activity during the initial training phase, i.e., the skill acquisition, followed by a reduction in cortical activity and an increased activity in subcortical regions such as the basal ganglia and the cerebellum with chronic training. In line with manual skill learning data, acute balance training increased occipital and parietal cortical activity [75], dorsolateral prefrontal cortex activity [76] and fronto-parietal network connectivity [33]. The dorsolateral prefrontal cortex is likely recruited when learning a new skill, because it plays a role in decision making and working memory, and it allows for the inhibition of unwanted responses, so that perturbation-specific responses can be executed without delays [76]. Contrary to the expected upregulation of cortical activity following acute balance training, a decreased coherence between relevant muscle synergies and the left paracentral lobule was reported in young individuals [74]. This decrease in coherence was interpreted as a response to short-term motor skill acquisition, which is associated with reduced activity in primary motor areas [88]. After balance training, less coherent or smaller neural populations were assumed to be needed to perform the same task [74]. In contradiction to and not supported by the results of the studies mentioned above, this finding would indicate a very rapid automatization of the balance task occurring following 30 min of balance board standing in young individuals. No studies examined the acute adaptations in cortical activity by imaging methods in older individuals.

Following chronic balance training, in line with manual skill learning, a reduction in cortical and an increase in subcortical activity are expected due to an increased automatization of the task. Indeed in young individuals, chronic balance training showed increased connectivity in the basal ganglia, indicating higher automaticity of task execution and subcortical involvement [68]. Also, in older individuals, chronic balance training reduced prefrontal cortex activity [36], as well as activity in motor, premotor, and multisensory vestibular areas [40] and the supplementary motor area [34]. These areas contribute to postural control and have been shown to reveal age-specific overactivation during the simulation and execution of motor tasks [38, 40]. Reduced activity in these areas may therefore suggest that balance training is able to reverse the age-related cortical over-activation and reverse the age-related shift in the neural control of posture. Also, following 6 weeks of slackline training, reductions in functional connectivity, as assessed by resting-state fMRI, were observed only in a subgroup of responders [35]. These reductions in functional connectivity in older individuals are again in line with previous manual motor skill learning studies and could reflect an increased efficiency of neural systems [89]. However, not all studies reported reductions in activity with chronic balance training, as one study reported no adaptations in resting-state cortical activity assessed by EEG [59]. It is possible that the specificity of the adaptations in cortical activity could explain the lack of results in this study since the cortical activity was assessed at rest.

While it is clear that both young and older individuals adapt cortical activity following balance training, the time course of these adaptations in older individuals is still unclear because no study has yet investigated the effects of acute balance training on cortical activity using imaging. Studies examining chronic balance training in both young and older individuals are in line with manual skill learning studies: reductions in cortical and increases in subcortical structures [14].

#### Structural Brain Adaptations

Acute structural adaptations in gray and white matter occurred rapidly, following just one or two 45-min balance training sessions in young individuals. These acute balance training interventions increased cortical thickness in the primary motor areas representing the foot and trunk [22] and increased gray matter volume in the frontal and parietal brain areas [37]. These structural changes in response to balance training occurred parallel to improvements in balance performance, indicating that these rapid structural adaptations are also functionally relevant. Interestingly, the rapid initial gray matter increases in sensorimotor-related areas decreased in the later learning phase, while the gray matter volume in the prefrontal cortex continuously increased.

No study has yet reported structural brain adaptations in response to acute balance training in older individuals. However, chronic balance training increased cortical thickness [17] and decreased hippocampal brain volume [79] and putamen volume [17]. Several balance training studies reported no changes in hippocampal, frontal lobe, cerebellar, and basal ganglia volumes in older individuals [35, 58, 61]. An age-related decline in brain volume could explain this lack of structural adaptation. It is possible that in older individuals, balance training is necessary to maintain brain volumes of structures that are relevant for motor and postural control. Without balance training, there would be age-typical reductions in these brain volumes. However, these results must be interpreted cautiously regarding the role that balance training might play in adaptations in brain volume, because there is emerging evidence, suggesting that this form of neural adaptation may be limited in humans [90–92].

#### Adaptations in Neurochemical Blood Markers

BDNF seems to mediate activity-dependent synaptic plasticity, and BDNF protein concentration is a biomarker that captures treatment effects and provides information on neural adaptation [34]. Improved balance performance after 20–36 balance training sessions over 10–12 weeks of balance training in older individuals was accompanied by increased BDNF levels [34, 62, 81]. However, no adaptations in BDNF level were reported after 36 sessions of Wii fit balance board training over 12 weeks. This lack of adaptation in BDNF level might reflect either a difference in intensity between the interventions or might be caused by a difference in the type of muscle contractions, where classic balance training involves more concentric and eccentric muscle work. In contrast, virtual balance training involves more isometric muscle activity [34]. Furthermore, the reliable determination of BDNF can be difficult [93].

### Limitations and Future Directions

While we were able to perform an omnibus meta-analysis on the data included in this review, the high level of heterogeneity in the training tasks, training duration, training frequency, balance performance measure, and measures of neural adaptation, reduce the reliability of these results. Also, funnel plot asymmetry indicated a publication bias which should be considered so that the effects are not overestimated. Second, there is a lack of short-term (1–3 training sessions) quantitative data available, making it impossible to quantitatively analyze the dose response effects of balance training on neural measures. Third, despite evidence that the adaptations in response to balance training are highly specific, motor conditions under which balance performance and neural adaptation were also assessed varied. Only 10 out of the 38 included studies examined the effects of balance training on the trained balance task (Fig. 2). We, therefore, recommend that future studies examine the effects of balance training on balance skills in the trained and non-trained balance task. We draw special attention to a lack of short-term and long-term retention in the trained and the non-trained transfer task. Markers of neural adaptation should be examined at rest, during the trained task, and during the non-trained transfer task, before and after acute and chronic balance training periods. While it appears that chronic balance training is not necessary for balance skill acquisition, it remains unclear whether longer training periods are instrumental for retaining the practiced balance skill and for its transfer to unpracticed balance skills. Future studies should examine whether training duration or session number is associated with skill retention. Establishing such an association seems of great importance, considering that balance training interventions are effective to reduce falls by ~ 25%, but we do not understand the mechanisms of how such protective effects emerge [8]. Fourth, rapid structural adaptations and adaptations in cortical activity assessed by imaging were only investigated and reported in young individuals. However, chronic balance training increased cortical thickness and gray matter volume in young and older individuals. Based on the available data, it is not possible to determine if age affects the time course of neural adaptation associated with balance skill learning. In order to understand the time course over which neural adaptation occurs, we recommend future studies to assess the neural correlates of postural control not only after several weeks of training, but also after shorter time periods and at multiple time points throughout the study. Fifth, this review included data from young individuals as a reference for the data from older individuals. However, the effects of balance training with respect to injury prevention or other aspects of balance training in young individuals were beyond the scope of the current review and should be examined in future studies.

## Conclusion

In conclusion, we systematically meta-reviewed the effects of balance training on the neural correlates of balance learning. The meta-analysis of 17 studies revealed that balance training improves neural measures overall. However, neither age nor training duration moderated these effects. Systematic synthesis, including 42 studies, showed that balance training rapidly improves balance performance in both young and older individuals. These changes are task-specific and are independent of the number of training sessions. Of the five types of neural correlates examined, changes in only spinal excitability seemed to differ between age groups. However, age and training dose in terms of duration did not moderate the effects of balance training on the changes in any of the neural correlates. The behavioral and neural mechanisms of strong task-specificity and the time course of skill retention remain unclear and require further studies in young and older individuals.

## Supplementary Information

**Additional file 1: Figure S1.** Full electronic search syntax used in PubMed. **Figure S2.** Forest plot showing the effects of balance training interventions on balance performance in trained and transfer tasks in 540 participants, 18 studies, and 60 outcomes (*g* = 0.96, 95% CI 0.647–1.28, *p* < 0.0001, *I*^2^ = 71%). These data are shown for sake of orientation, as the current review focused on neural not the behavioral outcomes of balance training. These data demonstrate that balance learning improved selected balance outcomes in the studies that were included in the current review and analyzed for neural correlates of balance training. Positive effect sizes denote favorable effects of balance training on balance performance. SPPB: short physical performance battery, *CoP* Center of pressure, *POMA* Performance-oriented mobility assessment, *ML* Medio-lateral, *GRF* Ground reaction force, *SEBT* Star excursion balance task. **Figure S3.** Funnel plot for studies included in the meta-analysis (*n* = 17) examining the effects of balance training on neural adaptation outcomes. Test for Funnel plot asymmetry: *z* = 3.53, *p* < 0.001. Limit Estimate *b* = − 2.142 (CI − 3.632, − 0.6514) indicates publication bias. **Figure S4.** Plot of influence diagnostics for studies examining the effects of balance training on neural adaptation. The red symbol denotes an influential case. The removal of this study in the sensitivity analyses did not significantly affect the subgroup meta-analyses. Panel labels refer to specific tests of influence diagnostics [51]. **Table S1.** Multi-variable meta-regression model for neural adaptation outcome measure with training duration and population as moderators. **Table S2.** PEDro Scale rating of included studies.

## Data Availability

The data sets generated and/or analyzed in this systematic review and meta-analysis are available from the corresponding author upon reasonable request.

## Abbreviations

AO: Action observation
AO + MI: A combination of motor imagery and action observation
AP: Anteroposterior
AT: Aerobic training
BBS: Berg Balance Scale
BDNF: Brain-derived neurotrophic factor
BT: Balance training
CD: Patients with cerebellar degeneration
CI: Confidence interval
CON: Control
CoP: Center of pressure
CT: Control, comparison task
DT: Dual task
EEG: Electroencephalography
EC: Eyes closed
EO: Eyes open
fMRI: Functional magnetic resonance imaging
fNIRS: Functional near-infrared spectroscopy
H: Healthy individuals
Hmax/Mmax: Maximal H-reflex to maximal M-wave ratio
HO: Healthy older individuals
Hslope and MEPslope: Slope of input–output relation H-reflex and MEP
HY: Healthy young individuals
ICF: Intracortical facilitation
LICI: Long intracortical inhibition
MEP: Motor-evoked potential
MI: Motor imagery
ML: Medio-lateral
MRI: Magnetic resonance imaging
MVC: Maximal voluntary contraction
QF: Quadriceps femoris muscle
PD: Parkinson’s disease patients
PFC: Prefrontal cortex
PEDro: Physiotherapy evidence database
POMA: Performance-oriented mobility assessment
PRISMA: Preferred Reporting Items for Systematic Reviews and Meta-Analysis
RMS: Root-mean-squared
ROI: Region of interest
SICI: Short intracortical inhibition
SE: Standard error
SOL: Soleus muscle
SPPB: Short physical performance battery
ST: Strength training
TA: Tibialis anterior muscle
TMS: Transcranial magnetic stimulation
TUG: Timed up and go
VR: Virtual reality
Wk, wks: Week(s)
WM: White matter
