# Supplementary material for: Neural Correlates of Balance Skill Learning in Young and Older Individuals: A Systematic Review and Meta-analysis
Source: Sports Med Open. 2024 Jan 7;10:3. doi: 10.1186/s40798-023-00668-3 (PMC10772137; doi:10.1186/s40798-023-00668-3)
Supplement: Supplementary file 1 — Additional file 1: Figure S1. Full electronic search syntax used in PubMed. Figure S2. Forest plot showing the effects of balance training interventions on balance performance in trained and transfer tasks in 540 participants, 18 studies, and 60 outcomes (g = 0.96, 95% CI 0.647–1.28, p < 0.0001, I2 = 71%). These data are shown for sake of orientation, as the current review focused on neural not the behavioral outcomes of balance training. These data demonstrate that balance learning improved selected balance outcomes in the studies that were included in the current review and analyzed for neural correlates of balance training. Positive effect sizes denote favorable effects of balance training on balance performance. SPPB: short physical performance battery, CoP Center of pressure, POMA Performance-oriented mobility assessment, ML Medio-lateral, GRF Ground reaction force, SEBT Star excursion balance task. Figure S3. Funnel plot for studies included in the meta-analysis (n = 17) examining the effects of balance training on neural adaptation outcomes. Test for Funnel plot asymmetry: z = 3.53, p < 0.001. Limit Estimate b = − 2.142 (CI − 3.632, − 0.6514) indicates publication bias. Figure S4. Plot of influence diagnostics for studies examining the effects of balance training on neural adaptation. The red symbol denotes an influential case. The removal of this study in the sensitivity analyses did not significantly affect the subgroup meta-analyses. Panel labels refer to specific tests of influence diagnostics [51]. Table S1. Multi-variable meta-regression model for neural adaptation outcome measure with training duration and population as moderators. Table S2. PEDro Scale rating of included studies. [file 40798_2023_668_MOESM1_ESM.docx]

Supplementary figure 1. Full electronic search syntax used in PubMed

**Supplementary figure 2.** Forest plot showing the effects of balance training interventions on balance performance in trained and transfer tasks in 540 participants, 18 studies, and 60 outcomes (g= 0.96, 95% CI: 0.647-1.28, p<0.0001, I^2^=71%). These data are shown for sake of orientation, as the current review focused on neural not the behavioral outcomes of balance training. These data demonstrate that balance learning improved selected balance outcomes in the studies that were included in the current review and analyzed for neural correlates of balance training. Positive effect sizes denote favorable effects of balance training on balance performance. SPPB: short physical performance battery, CoP: Center of Pressure, POMA: performance-oriented mobility assessment, ML: medio-lateral, GRF: ground reaction force, SEBT: Star excursion balance task.

**Supplementary figure 3.** Funnel plot for studies included in the meta-analysis (n=17) examining the effects of balance training on neural adaptation outcomes. Test for Funnel plot asymmetry: z=3.53, p<0.001. Limit Estimate b = -2.142 (CI: -3.632, -0.6514) indicates publication bias.

**Supplementary figure 4.** Plot of influence diagnostics for studies examining the effects of balance training on neural adaptation. The red symbol denotes an influential case. The removal of this study in the sensitivity analyses did not significantly affect the subgroup meta-analyses. Panel labels refer to specific tests of influence diagnostics [51].

**Supplementary Table 1.** Multi-variable meta-regression model for neural adaptation outcome measure with training duration and population as moderators.

Model: ES ~ Population + Duration

Number of studies = 17

Number of outcomes = 62 (min = 1 , mean = 3.65 , median = 2 , max = 13 )

Rho = 0.8

I^2^ = 84%

Tau^2^ = 0.93

Estimate SE t dfs P(|t|>) 95% CI.L 95% CI.U Sig

1 X.Intercept. 0.362 0.486 0.743 12.97 0.470 -0.689 1.41

2 PopulationHY -0.017 0.486 -0.036 12.97 0.972 -1.069 1.03

3 DurationChronic 0.470 0.311 1.512 5.98 0.181 -0.292 1.23

Signif. codes: < .01 *** < .05 ** < .10 *

Note: If df < 4, do not trust the results

Reference group: HO (for population) and acute (for duration).

The number of studies according to population: HY: 8; HO: 9

The number of studies according to Duration: Acute: 1; Chronic: 16

Supplementary table 2. PEDro Scale rating of included studies

| **Study** | **Randomization** | **Concealed Allocation** | **Comparable group baselines** | **Blinding of all subjects** | **Blinding of all therapists** | **Blinding of all assessors** | **Dropout <15%** | **Intention to treat analysis** | **Between-group statistics** | **Point and Variance Data** | **Total score** |
| --- | --- | --- | --- | --- | --- | --- | --- | --- | --- | --- | --- |
| Adcock 2020 [59] | N | N | N | N | N | N | Y | N | N | Y | 2/10 |
| Adcock 2020 [58] | Y | N | Y | N | N | N | Y | Y | Y | Y | 6/10 |
| Alizadehsaravi 2022 [16] | N | N | N | N | N | N | Y | N | N | Y | 2/10 |
| Bakker 2021 [39] | Y | N | Y | N | N | N | Y | Y | Y | Y | 6/10 |
| Beck 2007 [30] | Y | N | Y | N | N | N | Y | Y | Y | Y | 6/10 |
| Behrens 2015 [64] | Y | N | Y | N | N | N | Y | Y | Y | Y | 6/10 |
| Burciu 2013 [60] | N | N | N | N | N | N | Y | Y | Y | Y | 4/10 |
| Čekanauskaitė 2020 [82] | Y | N | Y | N | N | N | Y | Y | Y | Y | 6/10 |
| Chen 2011 [70] | N | N | Y | N | N | N | Y | Y | Y | Y | 5/10 |
| Chen 2022 [78] | Y | N | Y | N | N | N | N | Y | Y | Y | 5/10 |
| Eggenberger 2016 [36] | Y | N | Y | N | N | N | N | N | Y | Y | 4/10 |
| Esculier 2014 [32] | N | N | N | N | N | N | Y | Y | Y | Y | 4/10 |
| Freyler 2014 [27] | Y | N | Y | N | N | N | Y | Y | Y | Y | 6/10 |
| Giboin 2019 [68] | Y | N | Y | N | N | N | Y | Y | Y | Y | 6/10 |
| Giboin 2020 [63] | N | N | N | N | N | N | Y | N | N | Y | 2/10 |
| Gruber 2007 [28] | Y | N | Y | N | N | N | Y | Y | Y | Y | 6/10 |
| Im et al., 2023 [57] | N | N | N | N | N | N | N | N | N | Y | 1/10 |
| Keller 2012 [65] | Y | N | Y | N | N | N | Y | Y | Y | Y | 6/10 |
| Kubica 2019 [34] | Y | N | Y | N | N | N | Y | Y | Y | Y | 6/10 |
| Lauber 2011 [69] | N | N | Y | N | N | N | Y | Y | Y | Y | 5/10 |
| Lauber 2021 [72] | Y | N | Y | N | N | N | Y | Y | Y | Y | 6/10 |
| Magon 2016 [35] | Y | N | Y | N | N | N | Y | Y | Y | Y | 6/10 |
| Mouthon 2019 [73] | Y | N | Y | N | N | N | Y | Y | Y | Y | 6/10 |
| Nagamatsu 2016 [61] | Y | N | Y | N | N | N | N | Y | Y | Y | 5/10 |
| Nieman 2014 [80] | Y | N | Y | N | N | N | N | Y | Y | Y | 6/10 |
| Patel 2019 [77] | N | N | N | N | N | N | Y | N | N | Y | 2/10 |
| Penzer 2015 [74] | Y | N | Y | N | N | N | Y | Y | Y | Y | 6/10 |
| Peterson 2018 [76] | Y | N | Y | N | N | N | Y | Y | Y | Y | 6/10 |
| Rogge 2018 [79] | Y | N | Y | N | N | N | Y | Y | Y | Y | 6/10 |
| Ruffieux 2017 [66] | Y | N | Y | N | N | N | Y | Y | Y | Y | 6/10 |
| Ruffieux 2018 [40] | Y | N | Y | N | N | N | Y | Y | Y | Y | 6/10 |
| Schubert 2008 [31] | Y | N | Y | N | N | N | Y | Y | Y | Y | 6/10 |
| Sehm 2014 [17] | N | N | N | N | N | N | Y | Y | Y | Y | 4/10 |
| Solianik 2022 [81] | Y | N | Y | N | N | N | Y | Y | Y | Y | 6/10 |
| Szymure 2020 [62] | Y | N | Y | N | N | N | Y | Y | Y | Y | 6/10 |
| Taube 2007 [29] | Y | N | Y | N | N | N | Y | Y | Y | Y | 6/10 |
| Taube 2020 [71] | Y | N | Y | N | N | N | N | Y | Y | Y | 6/10 |
| Taubert 2010 [37] | Y | N | Y | N | N | N | Y | Y | Y | Y | 6/10 |
| Taubert 2011 [33] | Y | N | Y | N | N | N | Y | Y | Y | Y | 6/10 |
| Taubert 2016 [22] | N | N | Y | N | N | N | N | Y | Y | Y | 4/10 |
| Ueta 2022 [56] | Y | N | Y | N | N | N | Y | Y | Y | Y | 6/10 |
| Zandvoort 2019 [75] | N | N | N | N | N | N | Y | N | N | Y | 2/10 |

Abbreviations: Y, yes; N, no
